# The burden of non-alcoholic steatohepatitis: A systematic review of health-related quality of life and patient-reported outcomes

**DOI:** 10.1016/j.jhepr.2022.100525

**Published:** 2022-06-15

**Authors:** Zobair Younossi, Priya Aggarwal, Ichhya Shrestha, João Fernandes, Pierre Johansen, Margarida Augusto, Sunita Nair

**Affiliations:** 1 Center for Liver Diseases and Department of Medicine, Inova Fairfax Hospital, Falls Church, VA, USA; 2 Inova Medicine, Inova Health System, Falls Church VA, USA; 3 DRG Abacus (Clarivate), Gurgaon, Haryana, India; 4 Novo Nordisk A/S, Søborg, Denmark; 5 Novo Nordisk Denmark A/S, Region North & West Europe, Ørestad, Denmark; 6 Novo Nordisk Ltd, Gatwick, UK; 7 DRG Abacus (Clarivate), Mumbai, Maharashtra, India

**Keywords:** NASH, Non-alcoholic steatohepatitis, Burden of disease, Health-related quality of life, Patient-reported outcomes, Symptoms, Comorbidities, Disease progression, AIS, Athens Insomnia Scale, BC, biopsy-confirmed, BDI-II, Beck Depression Inventory-II, CC, compensated cirrhosis, CD, cognitive debriefing, CE, concept elicitation, CHC, chronic hepatitis C, CLD, chronic liver disease, CLDQ, Chronic Liver Disease Questionnaire, CVD, cardiovascular disease, e1, excluded after screening title and abstract, e2, excluded after screening full text, ELF, enhanced liver fibrosis, EPHPP, Effective Public Health Practice Project, EQ-5D, EuroQol-5D, EQ-5D-5L, EuroQol-5D-5 level, F1–4, fibrosis stages 1–4, FSSG, frequency scale for the symptoms of gastro-oesophageal reflux disease, GERD, gastro-oesophageal reflux disease, GfK, Growth from Knowledge, GGT, gamma-glutamyl transpeptidase, GI, gastrointestinal, HADS, Hospital Anxiety and Depression Scale, HCC, hepatocellular carcinoma, HRQoL, health-related quality of life, i1, included to screen based on title and abstract, i2, included to screen full text, i3, total included studies after the full-text review stage for original report and 2021 search update, MCID, minimal clinically important difference, MCS, mental component summary, N/A, not available, NAFL, non-alcoholic fatty liver, NAFLD, non-alcoholic fatty liver disease, NAS, NAFLD activity score, NASH, non-alcoholic steatohepatitis, NFS, non-alcoholic fatty liver disease fibrosis score, NICE, National Institute for Health and Care Excellence, NIT, non-invasive test, NR, not reported, OR, odds ratio, PCS, physical component summary, PHAQ, Patient-Reported Outcome Measurement Information System Health Assessment Questionnaire, PRISMA, Preferred Reporting Items for Systematic Reviews and Meta-Analyses, PRO, patient-reported outcome, QD, once daily, QoL, quality of life, RCT, randomised controlled trial, SF-12, 12-item Short Form Health Survey, SF-36, Short Form-36, SF-6D, Short Form–6 Dimension, SG, standard gamble, SPAN, School Physical Activity and Nutrition, T2D, type 2 diabetes, VAS, visual analogue scale, WPAI, Work Productivity and Activity Impairment, WPAI:SHP, Work Productivity and Activity Impairment: Specific Health Problem

## Abstract

**Background & Aims:**

Non-alcoholic steatohepatitis (NASH) is associated with increased mortality and a high clinical burden. NASH adversely impacts patients’ health-related quality of life (HRQoL), but published data on the humanistic burden of disease are limited. This review aimed to summarise and critically evaluate studies reporting HRQoL or patient-reported outcomes (PROs) in populations with NASH and identify key gaps for further research.

**Methods:**

Medline, EMBASE, the Cochrane Library and PsycINFO were searched for English-language publications published from 2010 to 2021 that reported HRQoL/PRO outcomes of a population or subpopulation with NASH.

**Results:**

Twenty-five publications covering 23 unique studies were identified. Overall, the data showed a substantial impact of NASH on HRQoL, particularly in terms of physical functioning and fatigue, with deterioration of physical and mental health as NASH progresses. Prevalent symptoms, including fatigue, abdominal pain, anxiety/depression, cognition problems, and poor sleep quality, adversely impact patients’ ability to work and perform activities of daily living and the quality of relationships. However, some patients fail to attribute symptoms to their disease because of a lack of patient awareness and education. NASH is associated with high rates of comorbidities such as obesity and type 2 diabetes, which contribute to reduced HRQoL. Studies were heterogeneous in terms of diagnostic methods, population, outcomes, follow-up time, and measures of HRQoL/utility. Most studies were rated ‘moderate’ at quality assessment, and all evaluable studies had inadequate control of confounders.

**Conclusions:**

NASH is associated with a significant HRQoL burden that begins early in the disease course and increases with disease progression. More robust studies are needed to better understand the humanistic burden of NASH, with adequate adjustment for confounders that could influence outcomes.

**Lay summary:**

Non-alcoholic steatohepatitis (NASH) has a significant impact on quality of life, with individuals experiencing worse physical and mental health compared with the general population. NASH and its symptoms, which include tiredness, stomach pain, anxiety, depression, poor focus and memory, and impaired sleep, affect individuals’ relationships and ability to work and perform day-to-day tasks. However, not all patients are aware that their symptoms may be related to NASH. Patients would benefit from more education on their disease, and the importance of good social networks for patient health and well-being should be reinforced. More studies are needed to better understand the patient burden of NASH.

## Highlights

• NASH is associated with a mental and physical quality of life burden that worsens as the disease progresses.
• NASH impacts patients’ relationships and ability to work and perform daily activities.
• Patients do not always know that symptoms such as fatigue are related to NASH.
• Patients with NASH and other diseases, such as obesity, have worse quality of life than those with NASH alone.
• More robust quality of life studies are needed.

## Graphical abstract

**Figure:**
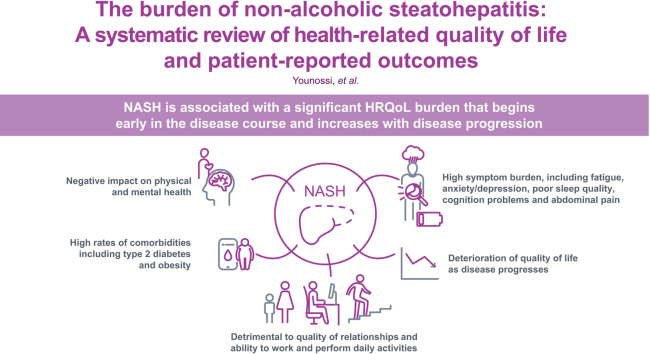

## Introduction

Non-alcoholic fatty liver disease (NAFLD) encompasses a spectrum of related liver disorders.^1^ It is defined by the presence of hepatic steatosis (accumulation of triglycerides in >5% of hepatocytes) in the absence of other causes of steatosis, including excess alcohol intake.[2], [3], [4] The earliest stage is termed simple steatosis or non-alcoholic fatty liver (NAFL), characterised by >5% hepatic steatosis without evidence of hepatocyte ballooning.^5^

In a subset of patients, simple steatosis can progress to non-alcoholic steatohepatitis (NASH; steatosis associated with lobular inflammation, hepatocellular ballooning, and fibrosis), and in its more advanced stages to cirrhosis and end-stage liver diseases.[3], [4], [5] NASH is strongly associated with obesity, type 2 diabetes (T2D), dyslipidaemia, and metabolic syndrome^6^ and confers an increased risk of hepatic complications (fibrosis progression, cirrhosis, and hepatocellular carcinoma [HCC]), extra-hepatic complications (*e.g.* solid organ malignancy, chronic kidney disease, and cardiovascular disease [CVD]), and mental health disorders (*e.g.* depression and anxiety).[7], [8], [9] NASH is the most rapidly increasing indication for liver transplantation in the US,^10^ and although NASH cirrhosis is currently the second leading cause of liver transplantation in the US, it is expected to become the leading cause between 2020 and 2025.^11^^,^^12^

The global prevalence of NAFLD is currently estimated to be ∼25%^7^^,^^11^ and is growing rapidly, fuelled by increasing rates of obesity and T2D.^4^^,^^11^ A recent meta-analysis estimated the overall prevalence of NASH in the general population as between 1.50% and 6.45%, and overall mortality incidence rates were 25.56 (range 6.29–103.80) per 1,000 person-years for NASH and 11.77 (range 7.10–19.53) for NAFLD.^11^

There are limited data on the impact of NASH on patients’ health-related quality of life (HRQoL). Available evidence suggests that, in addition to its clinical burden, NASH is associated with a high patient burden and adverse effect on patients’ HRQoL.[13], [14], [15] Understanding the impact of NASH and the effect of pharmacological treatments or lifestyle modification on HRQoL is important to inform future research on the development of patient-centred outcomes. The aim of this study was to summarise and critically evaluate studies that reported HRQoL or patient-reported outcomes (PROs) in populations with NASH and identify key evidence gaps.

## Materials and methods

A systematic literature review was conducted using Medline, EMBASE, the Cochrane Library and PsycINFO via the Ovid platform using predefined search strategies (Table S1). Hand searching was also used to identify relevant studies not captured in the electronic database search. The review was conducted in line with Cochrane Collaboration methodology^16^ and reported in line with PRISMA (Preferred Reporting Items for Systematic Reviews and Meta-Analyses).^17^ Further details on the review methodology are provided in the Supplementary methods.

The primary systematic review was undertaken on 17 February 2020 and an update performed on 6 January 2021. Eligible studies were full-text English-language publications published from 1 January 2010 to 6 January 2021 that reported HRQoL or PROs in populations with NASH or a subgroup with NASH within an NAFLD/NASH study, with or without comorbidities. NASH was defined based on individual study inclusion criteria. Full eligibility criteria are described in Table S2, and Table S3 summarises key features of the HRQoL and PRO instruments used across the studies.

First-round screening of titles and abstracts was followed by second-round full-text screening of shortlisted articles and data extraction of articles meeting the eligibility criteria. First- and second-round screening were performed by 2 independent researchers, and the final inclusion was verified by the project lead. Disagreements regarding eligibility were referred to a third party. Data extraction was performed using predesigned data extraction tables by an analyst with a 100% independent quality check, and disagreements were referred to a third party. Data extracted from each study included, but were not limited to, country/region, study design, baseline characteristics (including age, sex, BMI, method of diagnosis, and comorbidities), type of intervention (including dose, duration, and frequency), and outcomes reported (as shown in Table S1).

The quality of included observational studies was assessed using the Quality Assessment Tool for Quantitative Studies produced as part of the Effective Public Health Practice Project (EPHPP),^18^ which assesses the quality of 6 components (selection bias, design, confounders, blinding, data collection methods, and withdrawals/drop-outs) to assign a global rating for each study of ‘strong’ (no weak ratings for any of the listed criteria), ‘moderate’ (1 weak rating), or ‘weak’ (2 or more weak ratings).

Quality assessment (risk of bias) of randomised controlled trials (RCTs) was conducted using the 7-criteria checklist provided in Section 2.5.2 of the National Institute for Health and Care Excellence (NICE) single technology appraisal and highly specialised technologies evaluation user guide,^19^ which assesses the quality of studies and likelihood of selection, performance, attrition, and detection bias.

Two reviewers independently assessed the likelihood of bias, and any disagreements were resolved by discussion and/or additional referees.

## Results

**Fig. 1:**
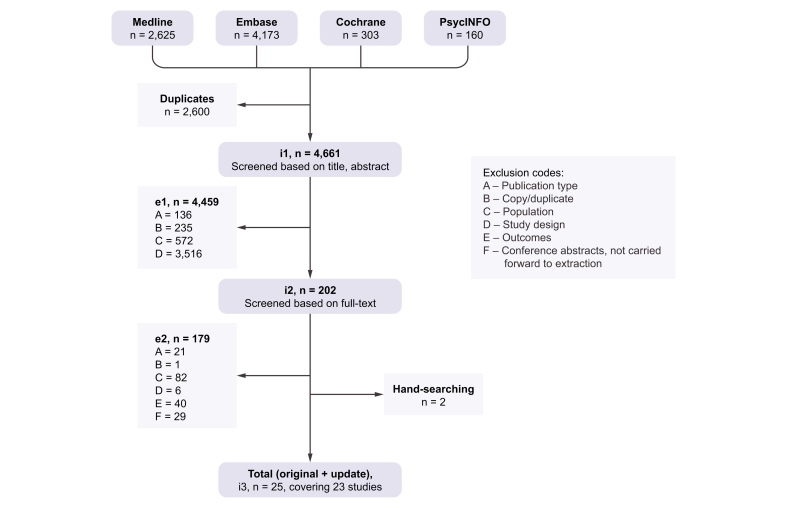
Flow diagram of publications included in and excluded from the systematic review. e1, excluded after screening title and abstract; e2, excluded after screening full text; i1, included to screen based on title and abstract; i2, included to screen full text; i3, total included studies after the full-text review stage for original report and 2021 search update.

The electronic database searches identified 7,261 citations, of which 2,600 were identified as duplicates and excluded. A further 4,459 were excluded based on title and abstract, and 179 during full-text screening, resulting in inclusion of 23 publications from the electronic database searches.[20], [21], [22], [23], [24], [25], [26], [27], [28], [29], [30], [31], [32], [33], [34], [35], [36], [37], [38], [39], [40], [41], [42] A further 2 studies meeting the eligibility criteria were identified from hand searching.^43^^,^^44^ The study flow of included and excluded publications is shown in Fig. 1.

Overall, 25 publications covering 23 unique studies in several countries were included (Table 1). Four studies were US-based, and 2 each were derived from the UK, Iran, and Japan. One study each originated from Germany, Korea, and Spain, whereas 10 studies described multinational data (Table 1). Six studies were interventional, 11 were cross-sectional, 2 were qualitative online tools/interviews, 3 were prospective, and 1 was retrospective in nature. An overview of the disease focus and instruments used across the identified studies, including a summary of the validity of liver- and NASH-specific instruments, is included in the Supplementary materials.

**Table 1:** **Publications meeting eligibility criteria for review**.

Study	Citation	Country	Quality^a^
**Quantitative studies**			

1	Alt *et al.*^20^	Germany	Moderate
2	Balp *et al.*^21^	Europe^b^	Weak
3	Chawla *et al.*^22^	USA	Moderate
4	Cook *et al.*^23^	Canada, Germany, UK, and USA	Moderate
5	Cook *et al.*^24^	UK and USA	Moderate
6	Doward *et al.*^25^	USA	Moderate
7	Elliott *et al.*^26^	UK	Moderate
8	Funuyet-Salas *et al.*^27^	Spain	Moderate
9	Geier *et al.*^28^	France, Germany, and USA	Moderate
10	Gholami *et al.*^29^	Iran	Moderate
11	Hattar *et al.*^30^	USA	Moderate
12	Huber *et al.*^31^	Germany, Spain, and UK	Moderate
13	Noto *et al.*^33^	Japan	Weak
14	Ock *et al.*^34^	Korea	Weak
15	O'Hara *et al.*^35^	Europe^b^ and USA	Moderate
16	Taketani *et al.*^36^	Japan	Moderate
17	Younossi *et al.*^38^	Global	Moderate
**Randomised controlled trials**			

18	Armstrong *et al.*^44^	UK	7/7
19	Nikroo *et al.*^32^	Iran	4/7
20	Sanyal *et al.*^43^	USA	7/7
21	Younossi *et al.*^37^	Canada and USA	5/7
22	Younossi *et al.*^41^	Global^c^	7/7
	Younossi *et al.*^39^		
	Younossi *et al.*^40^		
23	Younossi *et al.*^42^	Global^c^	1/7

a Quantitative studies were assigned a global rating of strong, moderate, or weak based on the quality of 6 components (selection bias, design, confounders, blinding, data collection methods, and withdrawals/drop-outs), whereas randomised controlled trials were assessed based on achievement of 7 criteria that assessed study quality and likelihood of selection, performance, attrition and detection bias.

b France, Germany, Italy, Spain, and the UK.

c A total of 27 countries across Asia, Australia, Europe, New Zealand, North America, and South America.

### Overall impact of NASH

Key data summarising the overall impact of NASH on patients’ HRQoL and an overview of reported symptoms of NASH and their impact are presented in Table 2, Table 3, respectively. Overall, patients with NASH had significantly reduced HRQoL (*p* <0.05) and health utility scores compared with the general population.^21^^,^^22^^,^^27^^,^^37^^,^^38^^,^^40^ Patients with NASH also reported worse mental functioning compared with a matched population with T2D^21^ and worse PRO scores related to physical domains *vs.* matched patients with chronic hepatitis C.^38^

**Table 2:** **Overall impact of NASH on patients’ HRQoL**.

Reference	Country/region	Population^a^ (N)	Data source	Key findings in patients with NASH/NAFLD
**Quantitative studies (cross-sectional)**				

Balp *et al.* 2019^21^	Europe (France, Germany, Italy, Spain, and UK)	NASH (184) Matched T2D (368) Matched general population^b^ (736)	National Health and Wellness Survey	• Compared with the matched general population, patients with NASH had the following: – Significantly worse SF-36v2 PCS (42.8 *vs.* 47.8; *p* <0.001) and MCS scores (39.2 *vs.* 45.2; *p* <0.001) – Significantly worse SF-6D (0.59 *vs.* 0.68; *p* <0.001) and EQ-5D utility scores (0.67 *vs.* 0.78; *p* <0.001) – Worse WPAI scores (more absenteeism [28.5 *vs.* 12.4%; *p* = 0.003], presenteeism [33.7 *vs.* 23.0%; *p* = 0.006], overall work impairment [49.2 *vs.* 30.8%; *p* <0.001], and activity impairment [48.0 *vs.* 32.6%; *p* <0.001]) • The cohort of patients with NASH also had significantly worse MCS (39.6 *vs.* 43.6; *p* = 0.003) and SF-6D utility scores (0.60 *vs.* 0.64; *p* = 0.002) than matched patients with T2D, but there was no difference between the groups for PCS and WPAI scores, or diagnoses of anxiety, depression, or sleep difficulties
Chawla *et al.* 2016^22^	US	Histology-proven NASH (79)	Single-centre, 1996–2000	• Compared with normative data from an age- and sex-matched US population, NASH was associated with the following: – Significant decrease in SF-36 PCS and MCS scores *vs.* age- and sex-matched US population (*p* <0.02 for both) along with significant reductions in all SF-36 components (*p* <0.05 for all) – Significant impairment in all CLDQ domains and reduced overall CLDQ score (*p* <0.0001 for all) *vs.* normative data from healthy controls
Cook *et al.* 2019^23^	Canada, Germany, UK, and USA	Confirmed or suspected NASH with F2 or F3 fibrosis stage (166)	Patient online bulletin boards and in-depth telephone interviews	• The overall mean (SD) EQ-5D-5L utility score was 0.81 (0.17) • Pain/discomfort and anxiety/depression were the most affected domains, with moderate to extreme problems reported in 37 and 26% of patients, respectively • Low impact of disease on self-care, mobility, and day-to-day working activities
Funuyet-Salas *et al.* 2020^27^	Spain	NASH (291) NAFL (201)	Twelve hospitals in 6 autonomous regions of Spain^d^	• Patients with NASH and NAFL with significant fibrosis had worse QoL by some dimensions of the SF-12 than the general Spanish population – NASH: physical functioning, role–physical, general health, vitality, role–emotional, mental health, and PCS (all *p* <0.001) – NAFL: physical functioning (*p* <0.001), general health (*p* <0.009), and PCS (*p* <0.001) • An interaction between NASH and social support was found in vitality (*p* = 0.05), activity (*p* = 0.005), anxiety (*p* = 0.04), and denial (*p* = 0.04) – Patients with NASH had less vitality, less activity, more anxiety, and more denial (all *p* <0.0001) when they perceived less social support • By relevant effect sizes (medium or large), patients with NASH with low social support had worse SF-36 (all domains except bodily pain) and CLDQ-NAFLD (all domains except abdominal symptoms and worry) QoL scores than those with high social support • In mental health, patients with low social support had higher scores in anxiety (HADS; *p* <0.001) and depressive symptoms (HADS and BDI-II; *p* <0.001 for both)
Geier *et al.* 2021^28^	France, Germany, and USA	Total NASH (1,216) **Pooled PRO cohort (299):** Biopsy-confirmed (160) Employed (143)	GfK (currently Ipsos) Disease Atlas Real-World Evidence Program (NASH-Atlas)	• Among 299 patients who completed the PRO survey, mean QoL scores were as follows: – CLDQ overall: 5.10 ± 1.43 – EQ-5D-5L utility score: 0.83 ± 0.21 – Absenteeism: 9.0 ± 22.5%^e^ – Presenteeism: 17.5 ± 19.9%^e^ – Overall work impairment: 24.7 ± 27.4%^e^ – Activity impairment: 30.7 ± 28.5%^e^
Ock *et al.* 2017^34^	Korea	NASH (N/A)	Korean general population	• Eight health states related to liver diseases (chronic HBV and HCV infections; NASH; liver cirrhosis; and HCC requiring partial hepatectomy, non-surgical treatments, liver transplantation, and palliative therapy) were assessed • As expected, the utility weights of health states decreased (worsened) as the severity of liver diseases increased • The highest VAS utility weights were HBV infection (0.640), followed by NASH (0.618), whereas the lowest was HCC that requires palliative therapy (0.17) • The highest SG utility weight was NASH (0.855), followed by chronic HBV infection (0.848), and the lowest was HCC that requires palliative therapy (0.40)
Younossi *et al.* 2019^38^	Global	NASH with advanced fibrosis^f^ (1,338) Matched CHC (1,338)	PRO database of participants from multinational clinical trials of selonsertib	• Participants with NASH had significantly worse PCS (mean 46.4 ± 9.5 *vs.* 50.0; *p* <0.0001) and health utility scores (0.68 ± 0.14 *vs.* 0.78; *p* <0.0001) *vs.* general population normative scores • PRO scores were substantially lower (worse) in patients with NASH *vs.* the matched cohort of patients with CHC in domains related to physical health (*p* <0.05 for physical functioning, bodily pain, general health, vitality, and PCS of SF-36, and fatigue of CLDQ; the mean impairment was between 2.5 and 7.8% of a PRO range size • In contrast, patients with CHC had lower scores in the mental health domain of SF-36 and emotional domain of CLDQ, and also a greater activity impairment score of WPAI (all *p* <0.05)
**Quantitative studies (prospective)**				
Huber *et al.* 2019^31^	Germany, Spain, and UK	NASH (210) NAFL (94)	European NAFLD registry	• The mean (SD) CLDQ overall score across all patients was 4.99 ± 1.2, with the lowest (worst) scores reported for fatigue (4.31 ± 1.6) and emotional functioning (1.93 ± 1.5), and the highest scores for activity (5.43 ± 1.4) and abdominal symptoms (5.33 ± 1.6) – This pattern was true for both cohorts of patients with NASH and NAFL • Women had significantly lower CLDQ scores than men (4.6 ± 1.3 *vs.* 5.3 ± 1.1; *p* <0.001) and significantly lower scores for all CLDQ subscales • Patients from the UK exhibited the lowest mean CLDQ overall score (4.7 ± 1.3)
Hattar *et al.* 2011^30^	US	Children (8–16 years) with the following: Biopsy-proven NASH and obesity (20) Obesity and no liver disease (20) Lean and no liver disease (17)	NR	• Children with both NASH and obesity reported a lower (worse) mean physical activity score (SPAN questionnaire) *vs.* both the obese and lean control groups (1.3 *vs.* 2.3 *vs.* 2.4, *p* <0.05)
**Qualitative studies**				

Doward *et al.* 2020^25^	US	NASH: CE interviews (23) CD interviews (20)^c^	Semistructured interviews conducted with patients with NASH in Virginia, USA	• Key HRQoL impacts in patients with NASH included impaired physical functioning, reduced ability to conduct daily living tasks, reduced quality of relationships, low mood, anxiety, and self-consciousness
**Interventional studies**				

Younossi *et al.* 2018^37^	Canada and USA	Biopsy-proven NASH with stage F2/F3 fibrosis (72)	RCT (NCT02466516)	• At baseline, physical health-related PROs of patients with NASH were lower than established general population levels, regardless of fibrosis stage (F3: *p* <0.05 for physical functioning, role-physical, general health, and PCS of SF-36; F2: *p* <0.05 for general health and PCS of SF-36) • Health-related work productivity and activity impairment scores at baseline were significantly greater than 0 (*p* <0.05 except for absenteeism)
Younossi *et al.* 2019^40^	Global	Biopsy-proven NASH with advanced fibrosis^f^ who completed PRO questionnaires^g^ (1,667)	Two RCTs (NCT03053050 and NCT03053063)	• In both groups of patients with bridging fibrosis (n = 797) and compensated cirrhosis (n = 870), mean SF-36 PRO scores (physical functioning, bodily pain, general health, role–emotional, and PCS) and EQ-5D and SF-6D utility scores were significantly lower (*p* <0.01) than those of the general population – Patients with cirrhosis also had significantly worse SF-36 role–physical and social functioning scores than the general population (*p* <0.01) – Patients with F4 fibrosis had score reductions of 4.4–12.9% in 6 of 8 SF-36 domains, and patients with F3 fibrosis had score reductions of 3.9–11.7% in 4 of 8 domains

BDI-II, Beck Depression Inventory-II; CD, cognitive debriefing; CE, concept elicitation; CHC, chronic hepatitis C; CLDQ, Chronic Liver Disease Questionnaire; EQ-5D, EuroQol-5 Dimension; EQ-5D-5L, EQ-5D-5 level; F1–4, fibrosis stage 1–4; GfK, Growth from Knowledge; HADS, Hospital Anxiety and Depression Scale; HCC, hepatocellular carcinoma; HRQoL, health-related quality of life; NAFL, non-alcoholic fatty liver; NAFLD, non-alcoholic fatty liver disease; NASH, non-alcoholic steatohepatitis; MCS, mental component summary; N/A, not available; NR, not reported; PCS, physical component summary; PHAQ, Patient-Reported Outcome Measurement Information System Health Assessment Questionnaire; PRO, patient-reported outcome; QoL, quality of life; RCT, randomised controlled trial; SF-6D, Short Form–6 Dimension; SF-12, 12-item Short Form Health Survey; SF-36, 36-Item Short Form Health Survey; SG, standard gamble; SPAN, School Physical Activity and Nutrition; T2D, type 2 diabetes; VAS, visual analogue scale; WPAI:SHP, Work Productivity and Activity Impairment: Specific Health Problem.

a NASH was defined based on individual study inclusion criteria.

b Representative sample of general population with varying health status.

c Eight participants took part in both groups.

d Andalusia, Cantabria, Castile and Leon, Catalonia, Madrid, and Valencia.

e In the subgroup of patients who completed the WPAI:SHP questionnaire (n = 141).

f Bridging fibrosis or compensated cirrhosis.

g SF-36, EQ-5D, CLDQ-NASH, and WPAI:SHP.

**Table 3:** **NASH symptoms and their impact on patients’ HRQoL**.

Reference	Country/region	Population (N)	Data source	Key findings in patients with NASH/NAFLD
**Quantitative studies (cross-sectional)**				

Balp *et al.* 2019^21^	Europe (France, Germany, Italy, Spain, and UK)	NASH (184) Matched T2D (368) Matched general population^a^ (736)	National Health and Wellness Survey	• Compared with the matched general population, patients with NASH had more diagnoses of anxiety (*p* <0.001), depression (*p* <0.001), and sleep difficulties (*p* = 0.025)
Cook *et al.* 2019^23^	Canada, Germany, UK, and USA	Confirmed or suspected NASH with F2 or F3 fibrosis stage (166)	Patient online bulletin boards and in-depth telephone interviews	• Patients were often unable to attribute their symptoms to NASH or other comorbid conditions • The most common reported symptoms, not attributed to their liver condition, were fatigue/tiredness (71%), being obese/overweight (62%), and abdominal pain (44%) • When asked about fatigue, 14% were unsure which of their health conditions contributed to this symptom and a further 14% did not associate fatigue with being a symptom of their liver condition
Geier *et al.* 2021^28^	France, Germany, and USA	Total NASH (1,216) Pooled PRO cohort (299): Biopsy-confirmed (160)	GfK (currently Ipsos) Disease Atlas Real-World Evidence program (NASH-Atlas)	• At first diagnosis of NASH (total population), symptoms were reported by 30.5% of patients. Key symptoms reported were fatigue (17.4%), abdominal bloating/swelling (13.7%), abdominal pain/discomfort (12.7%), and malaise (11.7%). Other symptoms were weight gain (10.6%), sleep apnoea/disturbance (7.1%), pruritus (5.3%), and jaundice (4.8%) • At the time of data collection, 58.0% of patients were symptomatic and had experienced symptoms for 27.0 ± 24.5 months • Of those in the pooled PRO cohort, 206 (68.9%) overall and 112 (70.0%) of biopsy-confirmed patients reported experiencing various symptoms at time of study. Incidence of symptoms (%) in pooled and biopsy-confirmed patients, respectively, was as follows^c^: – Fatigue: 38.5/37.5 – Malaise: 24.4/25.0 – Abdominal bloating: 26.1/25.6 – Weight gain: 14.4/19.4 – Abdominal pain: 15.0/13.1 – Sleep apnoea: 10.7/11.3 – Pruritus: 6.7/6.3 – Jaundice: 5.0/5.0 • Symptomatic patients had the following: – Significantly worse impairment *vs.* asymptomatic patients for CLDQ overall score and all CLDQ domain scores (*p* <0.05) – Lower (worse) mean EQ-5D-5L utility scores than asymptomatic patients – Greater impairment (among employed participants; n = 143) in all dimensions of the WPAI:SHP, reaching significance for presenteeism, overall work impairment, and activity impairment (all *p* <0.05) • In addition, a higher proportion of symptomatic patients reported problems across all EQ-5D-5L health dimensions compared with asymptomatic patients
**Qualitative studies**				

Cook *et al.* 2019^24^	UK and USA	NASH, fibrosis stages F1–F3 (16)	Physician referrals	• Patients did not spontaneously report any symptoms that they could directly associate with NASH • However, after probing, they acknowledged symptoms but did not necessarily associate them with NASH (fatigue, overweight, itching, sleeping problems, weakness/lethargy, anxiety, depression, flu-like symptoms, pain, and weight loss) • Fatigue (or daytime tiredness) was the most reported symptom • Patients reported that fatigue impacted their family relationships, work performance, and ability to complete daily tasks and maintain personal hygiene
Doward *et al.* 2020^25^	US	NASH: CE interviews (23) CD interviews (20)^b^	Single-centre (semistructured CE/CD interviews)	• Key symptoms reported in CE interviews were fatigue (78.3%), upper right abdominal pain (60.9%), cognition problems (impaired memory [56.5%] and reduced focus [47.8%]), and poor sleep quality (52.2%) • Symptoms of NASH impacted the following: – Physical functioning (impaired capacity to walk short distances) – Ability to conduct daily living tasks, for example, household chores/personal care – Quality of family relationships (*e.g.* pain/fatigue reduced ability to play with children/grandchildren or limited engagement in family life; negative impact on physical/emotional aspects of intimate relationships) – Sleep quality (reported for a range of issues including occurrence of pain) • Patients reported hiding their symptoms to reduce worry for family members • Ability to concentrate or complete work-related tasks impacted in patients in employment
**Interventional studies**				

Younossi *et al.* 2020^41^	Global	Biopsy-proven NASH with advanced fibrosis^d^ Total: 1,699 Pruritus: 447^e^ No pruritus: 1,222^e^ Fatigue: 549^e^ No fatigue: 1,121^e^	Two RCTs (NCT03053050 and NCT03053063)	• Clinically significant pruritus and fatigue^e^ were present in 27 and 33% of patients, respectively • Median pruritus and fatigue scores were 6 (IQR 4–7) and 4.8 (IQR 3.7–5.7), respectively • Patients with pruritus had demographic characteristics similar to those with fatigue but a higher prevalence of dermatologic comorbidities • Baseline PRO scores (all domains of SF-36, CLDQ-NASH, WPAI:SHP, SF-6D, and EQ-5D) were impaired in patients with pruritus (by up to -19% of a range size; all *p* <0.01) – Female sex, lower serum albumin, a history of depression, and nervous system and dermatologic comorbidities were associated with increased risk of pruritus (*p* <0.05) • Baseline PRO scores were also significantly impaired in patients with fatigue (mean up to -31% of a PRO range size in comparison with patients without fatigue; all *p* <0.01) – In multivariate analysis, predictors of fatigue included diabetes, history of depression or nervous system comorbidities, and lower serum albumin (*p* <0.05) • Patients with both fatigue and pruritus (n = 249) had the most profound impairment to PRO scores (mean impairment *vs.* patients with neither fatigue nor pruritus was between -5 and -37.5% of a PRO range size)

CD, cognitive debriefing; CE, concept elicitation; CLDQ, Chronic Liver Disease Questionnaire; EQ-5D, EuroQol-5 Dimension; EQ-5D-5L, EQ-5D-5 level; F1–4, fibrosis stages 1–4; GfK, Growth from Knowledge; HRQoL, health-related quality of life; NAFLD, non-alcoholic fatty liver disease; NASH, non-alcoholic steatohepatitis; PRO, patient-reported outcome; RCT, randomised controlled trial; SF-36, 36-item Short Form Health Survey; SF-6D, Short Form–6 Dimension; T2D, type 2 diabetes; WPAI:SHP, Work Productivity and Activity Impairment: Specific Health Problem.

a Representative sample of general population with varying health status.

b Eight participants took part in both groups.

c Data are presented in publication as a proportion of symptomatic patients. We have recalculated as a proportion of the full PRO cohort.

d Bridging fibrosis (F3) or compensated cirrhosis (F4).

e Presence of fatigue and pruritus were indicated by a score of 4 or less on the respective items of the CLDQ-NASH (scale range 1–7).

For example, in 1 study from Balp *et al.*^21^ that assessed 184 multinational patients with NASH who participated in the National Health and Wellness Survey, patients with NASH had significantly worse HRQoL than a matched general population cohort, shown by lower (worse) Short Form-36 (SF-36) physical component summary (PCS) and mental component summary (MCS) scores, lower Short Form–6 Dimension (SF-6D) and EuroQol-5D (EQ-5D) utility scores (*p* <0.001 for all), and higher rates of absenteeism (*p* = 0.003), presenteeism (*p* = 0.006), overall work impairment (*p* <0.001), and activity impairment (*p* <0.001). Patients with NASH also reported lower SF-36 MCS (*p* = 0.003) and SF-6D scores (*p* = 0.002) than a matched T2D cohort, but no significant differences were seen in terms of SF-36 PCS, EQ-5D, or Work Productivity and Activity Impairment (WPAI) scores.

Two studies, including a large database study that evaluated 1,338 patients with NASH, noted an adverse impact on physical but not mental function in patients with NASH compared with healthy individuals,^37^^,^^38^ whereas other studies reported deterioration in both physical and mental PRO domains compared with the general population.^21^^,^^22^^,^^27^^,^^40^

One study that assessed patients with NAFLD from 12 hospitals across Spain found an interaction between HRQoL and the level of social support received by patients with NASH from family, friends, their partner, or other significant persons.^27^ A significant interaction between NASH and social support was found in terms of vitality, activity, anxiety, and denial (all *p* ≤0.05), with worse SF-36, Chronic Liver Disease Questionnaire (CLDQ)-NAFLD, Hospital Anxiety and Depression Scale, and Beck Depression Inventory-II scores among patients who perceived low levels of social support. Interestingly, the same was not true among patients with non-NASH NAFLD, for whom no differences in activity, anxiety, and denial were present between those with high and those with low perceived social support; only vitality was lower in patients with low perceived social support.^27^

### Impact of NASH symptoms

Several studies evaluated symptoms of NASH and their impact on patients’ HRQoL (Table 3).^21^^,^[23], [24], [25]^,^^28^^,^^41^ Key symptoms reported across the studies in patients with NASH included fatigue/tiredness (38–78%) and abdominal pain (13–61%).^23^^,^^25^^,^^28^^,^^41^ Other reported symptoms included poor sleep quality, sleep apnoea, weakness/lethargy, anxiety/depression, weight gain, cognition problems (impaired memory and focus), abdominal bloating, pruritus, and jaundice.^21^^,^[23], [24], [25]^,^^28^^,^^41^ Patients reported that NASH and its associated symptoms impacted many aspects of their lives, including physical functioning, work performance, ability to perform daily living tasks, and the quality of family and personal relationships.^23^^,^^25^ Two studies reported greater impairment of PRO scores (SF-36, CLDQ, CLDQ-NASH, WPAI: Specific Health Problem [WPAI:SHP], and health utility instruments) in symptomatic *vs.* non-symptomatic patients.^28^^,^^41^

Notably, 2 studies reported that patients may not always directly associate their symptoms with NASH.^23^^,^^24^ A multinational survey-based study of 166 patients with confirmed or suspected NASH asked participants to indicate whether the origin of certain symptoms could be attributed to NASH or a comorbid condition.^23^ When asked about fatigue, for example, 14% of patients attributed this solely to other conditions, and a further 14% were unsure of what was causing their symptom. In another small study of 16 patients from the USA and UK, patients did not spontaneously report any symptoms that they could directly associate with NASH; symptoms were acknowledged after probing but were not necessarily linked to NASH.^24^

### Impact of disease severity

**Fig. 2:**
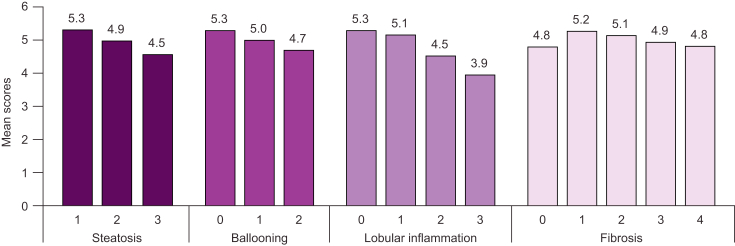
CLDQ scores according to NASH/NAFLD severity.^31^ Higher scores indicate better quality of life. CLDQ, Chronic Liver Disease Questionnaire; NAFLD, non-alcoholic fatty liver disease; NASH, non-alcoholic steatohepatitis.

The impact of disease severity on HRQoL scores is summarised in Table 4 and below. Overall, populations with NASH reported worse HRQoL than patients with simple steatosis.^26^^,^^27^^,^^31^ Among patients with NASH, more severe fibrosis and advanced stages of liver disease generally had a negative impact on patients’ HRQoL and health utility scores.^27^^,^^31^^,^^35^^,^^38^^,^^40^^,^^42^ For example, a study of over 300 patients from the European NAFLD Registry showed that patients with progressive NASH (indicated by higher steatosis histological score, more severe ballooning, and higher levels of lobular inflammation) exhibited worse CLDQ scores than patients with less advanced disease (Fig. 2).^31^ This was not consistent across all studies; for example, a single-centre study from the UK that assessed 224 patients with NAFLD did not find any significant difference in terms of difficulty with function (assessed using the Patient-Reported Outcome Measurement Information System Health Assessment Questionnaire [PHAQ]) between those who had pre-cirrhosis and those who had cirrhosis (*p* = 0.3),^26^ and 1 *post hoc* analysis of a phase II RCT by Younossi *et al.*^37^ did not show any differences in HRQoL between patients with NASH with F2 fibrosis (n = 25) and those with F3 (n = 47) fibrosis.

**Table 4:** **Impact of liver disease severity and progression on patients’ HRQoL**.

Reference	Country	Population (N)	Data source	Key findings
**Quantitative studies (cross-sectional)**				

Funuyet-Salas *et al.* 2020^27^	Spain	NASH (291) NAFL (201)	Twelve hospitals in 6 autonomous regions of Spain^a^	• NASH patients had worse scores for all SF-36 and CLDQ-NAFLD domains than those with NAFL (all *p* ≤0.03) • By relevant effect sizes (medium and large), patients with NASH and significant fibrosis scored lower (worse) than those without significant fibrosis in terms of SF-36 physical functioning, role–physical, and PCS (all *p* <0.001) and CLDQ-NAFLD overall score and all domains (all *p* <0.001) – Patients with NASH and significant fibrosis also showed higher (worse) scores in total depression by HADS and BDI-II (both *p* <0.001) • Patients with NASH, but not patients with NAFL, had less activity, more anxiety, and more denial (all *p* <0.0001) when they perceived less social support – Both groups had less vitality with lower perceived social support (*p* <0.0001) • When social support was high, there were no differences between patients with NASH and those with NAFL, but when social support was low, patients with NASH had lower scores than patients with NAFL in vitality (*p* <0.001) and activity (*p* <0.001)
O’Hara *et al.* 2020^35^	USA and EU5 (France, Germany, Italy, Spain, and UK)	NASH (3,754) Biopsy-confirmed NASH (1,619) Provided QoL data (767)	Multinational study (GAIN)	• Mean EQ-5D utility value decreased (worsened) with fibrosis status in all countries with the exception of France. Overall score ranged from 0.80 in early disease stages (F0–F2; n = 531) to 0.62 in advanced stages (F3–F4; n = 218), with an overall mean utility value of 0.75 • Mean CLDQ score was 4.9. There was a soft trend for CLDQ-NAFLD score decrease with fibrosis stage (5.2 *vs.* 4.4 for early [n = 513] *vs*. advanced [n = 210] disease stage, respectively), but this was not observed in all territories
Taketani *et al.* 2014^36^	Japan	Biopsy-proven NAFLD, including the following: NASH (83) NAFL (40)	Hepatology centres in the Japan Study Group of NAFLD	• The proportion of patients with an AIS score ≥6 (diagnostic of insomnia) was similar in the groups of patients with NAFL and NASH (25 *vs.* 29%; *p* = 0.8299) • FSSG score did not differ between the groups of patients with NAFL and NASH, with 25% GERD prevalence in each group
**Quantitative studies (prospective)**				

Elliott *et al.* 2013^26^	UK	NAFLD (224)	Single-centre	• Participants with progressive liver disease (NASH) had significantly more functional difficulty than those with simple steatosis (PHAQ median 18.75 [range 0–100] *vs.* 6.25 [0–93.5]; *p* <0.05) – There was no significant difference in the function of participants with NAFLD who had cirrhosis compared with those who had pre-cirrhosis (PHAQ scores median range 18.6 [0–75] *vs.* 12.5 [0–100]; *p* = 0.3)
Huber *et al.* 2019^31^	Germany, Spain, and UK	NASH (210) NAFL (94)	European NAFLD registry	• NASH was associated with a significantly lower CLDQ score compared with patients with NAFL (mean 4.85 *vs.* 5.31; *p* <0.01) – Except for abdominal symptoms and emotional function, patients with NASH scored significantly lower than those with NAFL on all CLDQ subscales (fatigue, systemic symptoms, activity, worry; all *p* <0.01) • Higher steatosis histological score resulted in lower mean CLDQ score (grade 3 *vs.* 1, 4.5 *vs.* 5.3; *p* <0.05) • Lower mean CLDQ scores were associated with more severe ballooning (grade 2 *vs.* 0, 4.7 *vs.* 5.3; *p* <0.05) and higher levels of lobular inflammation (grade 3 *vs.* 0, 3.9 *vs.* 5.3; *p* <0.001) • Advanced fibrosis and compensated cirrhosis (F3/F4; n = 127) exhibited a trend toward lower HRQoL (F3–4 *vs.* F0–2, 4.9 *vs.* 5.1; *p* = 0.07)
**Interventional studies**				

Sanyal *et al.* 2010^43^	US	Patients with NASH without diabetes (247) Placebo cohort (83) Vitamin E 800 IU QD (natural form) cohort (84) Pioglitazone 30 mg QD cohort (80)	RCT (NCT00063622)	• Vitamin E was associated with a significantly higher rate of improvement in NASH *vs*. placebo (*p* = 0.001), and both active treatments had a significant reduction in steatosis, lobular inflammation, and NAS *vs*. placebo (*p* <0.05) but no improvement in fibrosis scores (*p* >0.05) • Despite this, change in SF-36 PCS and MCS scores did not differ significantly between placebo (PCS: -0.3; MCS: 0.4) and vitamin E (PCS: 0.4; MCS: -0.5) or pioglitazone (PCS: -0.9; MCS: -1.9) (all *p* <0.05)
Younossi *et al.* 2018^37^	Canada and USA	Biopsy-proven NASH with stage F2/F3 fibrosis (72)	RCT (NCT02466516)	• There was no difference in baseline SF-36, CLDQ, and WPAI:SHP scores between patients with F2 (n = 25) and those with F3 (n = 47) fibrosis (all domains *p* >0.05) • However, patients with NASH who experienced ≥2 decrease (improvement) in NAS or ≥1-stage reduction in fibrosis showed significant improvements in their PROs (up to +15.5 points on a universal 0–100 PRO scale; *p* <0.05) • Up to 21.5% improvements in PROs (*p* <0.05) were noted in patients with ≥50% relative reduction in collagen, whereas patients with NASH with >17% increase in their collagen experienced PRO worsening (up to -13.9%, *p* <0.05)
Younossi *et al.* 2019^39^	Global	Biopsy-proven NASH with advanced fibrosis^b^ who completed CLDQ-NASH (1,667)	Two RCTs (NCT03053050 and NCT03053063)	• Patients with NASH with cirrhosis had significantly lower (worse) CLDQ-NASH scores *vs.* patients with bridging fibrosis without cirrhosis (*p* <0.015 for all domains except systemic)
Younossi *et al.* 2019^40^		Biopsy-proven NASH with advanced fibrosis^b^ who completed PRO questionnaires^c^ (1,667)		• Patients with F4 fibrosis had lower scores than those with F3 fibrosis in the following (all *p* <0.01): – CLDQ-NASH: total score and abdominal, activity, emotional, fatigue, and worry domains – SF-36: role–physical, bodily pain, and social functioning domains – EQ-5D • In multivariate analysis, independent predictors of higher (better) PRO scores in patients with advanced fibrosis included older age, male sex, Asian race, and location of enrolment (*p* <0.01) • Black race, current smoking, higher BMI, diabetes mellitus, comorbidities (gastrointestinal, musculoskeletal, nervous system, and psychiatric disorders), and having cirrhosis were predictive of lower (worse) PRO scores in patients with advanced fibrosis
Younossi *et al.* 2020^41^		Biopsy-proven NASH with advanced fibrosis^b^ (1,699)		• There was no difference between patients with bridging fibrosis and those with cirrhosis in terms of median pruritus score (*p* = 0.55) • Patients with bridging fibrosis had higher median fatigue scores (indicating less fatigue) than those with cirrhosis (*p* = 0.0028), but the difference did not meet the threshold of an MCID for domain scores of CLDQ-NASH
Younossi *et al.* 2021^42^	Global	Biopsy-proven NASH with advanced fibrosis^b^ Total: 2,154 Bridging fibrosis: 1,021 CC: 1,133	Four RCTs (NCT01672866, NCT01672879, NCT03053050, and NCT03053063)	• At baseline, markers of advanced disease (NITs: hepatic collagen ≥11.2%, FibroScan® ≥23.4 kPa, NAS ≥5, ELF ≥10.43, NFS ≥1.802, and FibroTest™ score of 0.54) were associated with significant impairment across multiple domains of the SF-36, EQ-5D, CLDQ-NASH, and WPAI:SHP PROs • During treatment, only the worry domain of CLDQ-NASH improved in the pooled cohort (*p* <0.0001); other PRO scores remained at their baseline level or worsened – Neither achieving the studies’ primary endpoint (≥1-stage fibrosis improvement without worsening of NASH, n = 209) nor improvement of fibrosis stage regardless of NASH (n = 264) was associated with improvement in PROs (all *p* >0.05 except for worry) • Patients who achieved a ≥2-point decrease (improvement) in NAS experienced greater improvement in general health than those who did not, as well as significant improvement in 4 of 6 domains and total score of the CLDQ-NASH (*p* <0.05) • During treatment, decreases (improvement) in NAS and NIT scores were associated with improved PRO scores, whereas increases (worsening) in NIT scores were associated with worsened PRO scores (*p* <0.05)

AIS, Athens Insomnia Scale; BDI-II, Beck Depression Inventory-II; CC, compensated cirrhosis; CLDQ, Chronic Liver Disease Questionnaire; EQ-5D, EuroQol-5 Dimension; ELF, enhanced liver fibrosis; F1–4, fibrosis stages 1–4; FSSG, frequency scale for the symptoms of gastro-oesophageal reflux disease; GERD, gastro-oesophageal reflux disease; HADS, Hospital Anxiety and Depression Scale; HRQoL, health-related quality of life; MCID, minimal clinically important difference; NAFL, non-alcoholic fatty liver; NAFLD, non-alcoholic fatty liver disease; NAS, NAFLD Activity Score; NASH, non-alcoholic steatohepatitis; NFS, non-alcoholic fatty liver disease fibrosis score; NIT, non-invasive test; PCS, physical component summary; PHAQ, Patient-Reported Outcome Measurement Information System Health Assessment Questionnaire; PRO, patient-reported outcome; RCT, randomised controlled trial; SF-36, 36-item Short Form Health Survey; QD, once daily; WPAI:SHP, Work Productivity and Activity Impairment: Specific Health Problem.

a Andalusia, Cantabria, Castile and Leon, Catalonia, Madrid, and Valencia.

b Bridging fibrosis or compensated cirrhosis.

c SF-36, CLDQ-NASH, EQ-5D, and WPAI:SHP.

**Fig. 3:**
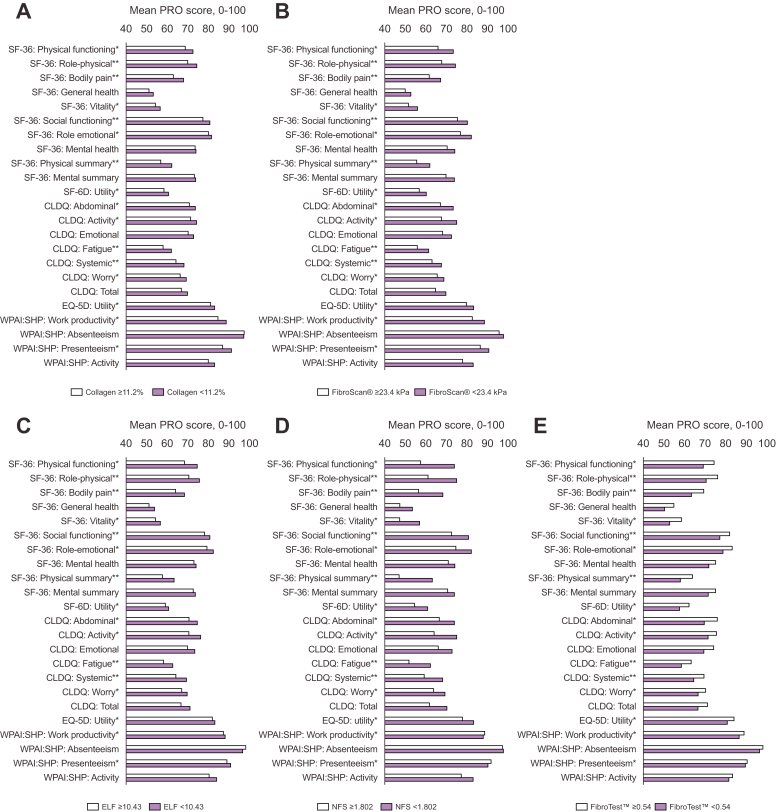
Mean baseline PRO scores in patients with NASH. By (A) baseline hepatic collagen, (B) transient elastography by FibroScan®, (C–E) baseline non-invasive test scores.^42^ ∗*p* <0.05, ∗∗*p* <0.01. CLDQ, Chronic Liver Disease Questionnaire; ELF, enhanced liver fibrosis; EQ-5D, EuroQol-5D; NASH, non-alcoholic steatohepatitis; NFS, non-alcoholic fatty liver disease fibrosis score; PRO, patient-reported outcome; SF-36, Short Form-36; SF-6D, Short Form–6 Dimension; WPAI:SHP, Work Productivity and Activity Impairment: Specific Health Problem.

Links between non-invasive markers of disease severity and HRQoL were evaluated in 2 studies.^37^^,^^42^ In a pooled analysis of over 2,000 patients who participated in 4 phase II/III clinical studies of simtuzumab or selonsertib *vs.* placebo, higher non-invasive test (NIT) scores (hepatic collagen ≥11.2%, FibroScan® ≥23.4 kPa, NAFLD activity score [NAS] ≥5, enhanced liver fibrosis score ≥10.43, NAFLD fibrosis score ≥1.802, and a FibroTest™ score of 0.54) were associated with impaired HRQoL.^42^ During treatment, decreases in NIT scores (indicating improvement) were associated with improved PRO scores and increases (worsening) with worsened PRO scores (*p* <0.05; Fig. 3).^42^ Similar findings were reported in a smaller analysis of 72 selonsertib-treated patients.^37^

Only 1 study evaluated the impact of pharmacological intervention *vs*. placebo on HRQoL. In the 96-week phase III PIVENS study, change in quality of life, assessed by SF-36 PCS and MCS scores, did not differ significantly between placebo and vitamin E or pioglitazone (all *p* >0.05), despite a significantly higher rate of improvement with vitamin E *vs.* placebo, and a significant reduction in NASH histological features and NAS with both active treatments *vs.* placebo (Table 4).^43^

### Comorbidities

The prevalence of comorbidities associated with NASH was high but varied widely across the studies (Table 5). Some of the most frequently reported comorbidities were obesity (35–96%),[20], [21], [22], [23], [24], [25]^,^^28^^,^^29^^,^^31^^,^^33^^,^^35^^,^^36^ diabetes (12–74%),[20], [21], [22], [23], [24], [25]^,^^28^^,^^29^^,^^31^^,^^33^^,^[35], [36], [37], [38], [39]^,^^44^ hypertension (27–76%),[20], [21], [22], [23]^,^^25^^,^^28^^,^^29^^,^^33^^,^[35], [36], [37]^,^^44^ hyperlipidaemia/dyslipidaemia (31–69%),^20^^,^^23^^,^^25^^,^^28^^,^^33^^,^^35^^,^^36^^,^^44^ and CVD (6–69%).^21^^,^^23^^,^^28^^,^^44^ Depression was the most frequently reported mental health comorbidity.^23^^,^^25^^,^^28^^,^^35^^,^^38^^,^^41^

**Table 5:** **NASH comorbidities and their impact on HRQoL**.

Reference and country	Population (N)	Data source	Incidence of key comorbidities, %					Other key findings
			Obesity^a^	Diabetes	Hypertension	Hyperlipidaemia/dyslipidaemia	CVD	
**Quantitative studies (cross-sectional studies)**								

Balp *et al.* 2019^21^ Europe (France, Germany, Italy, Spain, and UK)	NASH (184) T2D (368) General population^b^ (736)	National Health and Wellness Survey	46.7^c^	22.8	49.5	NR	69.0	
Chawla *et al.* 2016^22^ USA	NASH (79)		60	19	37	NR	NR	Significant reduction in SF-36 PCS score (37 *vs.* 45, *p* = 0.04) and CLDQ total score (4.1 *vs.* 5.1, *p* = 0.01) in patients with *vs.* without T2D
Cook *et al.* 2019^23^ Canada, Germany, UK, and USA	Confirmed or suspected NASH with F2 or F3 fibrosis stage (166)	Patient online bulletin boards and in-depth telephone interviews	68.7^c^	53.0^d^	48.2	43.4	11.4^e^	Other comorbidities included depression (15.7%), sleep apnoea (15.1%), joint/bone issues (12.7%), and muscle issues (6.0%)
Geier *et al.* 2021^28^ France, Germany, and USA	Total NASH: 1,216 BC: 786 (64.6%) BC cohort: No fibrosis: 55 (7.0%) F1: 175 (22.5%) F2: 278 (35.4%) F3: 211 (26.8%) F4: 47 (6.0%) Unknown: 20 (2.5%)	GfK (currently Ipsos) Disease Atlas Real-World Evidence program (NASH-Atlas)	Subgroup with recorded available data (n = 502)					Other reported comorbidities included sleep apnoea (14.2% of BC cohort, range 7.5–28.3% across F1 to F4 fibrosis stages) and depression (11.5% of BC cohort, range 9.4–12.0% across F1 to F4 fibrosis stages)
			47.6	59.2	48.2	40.4	9.3^e^	
			BC cohort					
			BC: 51.0 F1: 43.4 F2: 57.6 F3: 49.5 F4: 46.4	BC: 62.2 F1: 54.7 F2: 62.0 F3: 66.0 F4: 71.4	BC: 48.0 F1: 45.3 F2: 41.3 F3: 52.4 F4: 53.6	BC: 41.6 F1: 35.8 F2: 42.4 F3: 49.5 F4: 32.1	BC: 5.7^e^ F1: 3.8^e^ F2: 6.5^e^ F3: 6.8^e^ F4: 7.1^e^	
Gholami *et al.* 2018^29^ Iran	Suspected NASH (332)	Amol cohort health study	95.5^c^	11.7	29.2	NR	NR	Presence of metabolic syndrome (n = 144; 43.4%) negatively associated with 4 SF-12 subscales after adjustment for other variables: • Role limitations caused by physical problems (−14.05; *p* = 0.004) • Bodily pain (−7.37; *p* = 0.02) • Vitality (−7.72; *p* = 0.022) • Role limitations caused by emotional problems (−12.67; *p* = 0.005) Metabolic syndrome also had a borderline association with the general health and mental health subscales and the PCS (*p* <0.1) T2D was associated with worse mental health (*p* = 0.05) and MCS scores (*p* = 0.07) *vs.* no T2D, and history of hypertension was associated with worse vitality (*p* = 0.06) and MCS (*p* = 0.08)^g^*vs.* no history of hypertension
Noto *et al.* 2014^33^ Japan	Biopsy-proven NASH (171)	Single-centre (1995–2010)	Male					
			77.4^h^	25.8	32.3	34.4	NR	
			Female					
			59.0^h^	42.3	42.3	44.9	NR	
			Overall					
			69.6^h^	33.3	38.0	38.6	NR	
O’Hara *et al.* 2020^35^ USA and Europe (France, Germany, Italy, Spain, and UK)	Patients with NASH (3,754)	Multinational study (GAIN)	35	27	27	32	NR	Comorbidity rates showed a relatively similar trend across countries Depression was the most common mental and behavioural disorder, reported by 8% of patients overall and ranging from 10% in Italy to 6% in the USA Cardiovascular diseases (excluding hypertension) were most reported in Germany (18%) and least commonly reported in the USA (4%)
Taketani *et al.* 2014^36^ Japan	Biopsy-proven NAFLD, including the following: NASH (83) NAFL (40)	Hepatology centres in the Japan Study Group of NAFLD	72.3^h^	50.6	45.8	41.0	NR	Nearly 30% of patients with biopsy-proven NAFLD had insomnia, which was related to GERD and concentrations of GGT
Younossi *et al.* 2019^38^ Global	NASH with advanced fibrosis^a^ (1,338)	PRO database of participants from multinational clinical trials of selonsertib	NR	73.8	NR	NR	NR	Other comorbidities included cirrhosis (54.4%), anxiety (19.4%), and depression/mood disorders (25.3%); average BMI was 33.7 ± 6.5 Comorbidities associated with impairment of physical health (PCS in the lowest quartile) are as follows: • BMI (OR 1.058, 95% CI 1.036–1.081; *p* <0.0001) • T2D (OR 1.54, 95% CI 1.11–2.12; *p* = 0.0093) • Depression (OR 1.55, 95% CI 1.09–2.19; *p* = 0.014)
**Quantitative studies (prospective)**								

Huber *et al.* 2019^31^ Germany, Spain, and UK	NASH (210) NAFL (94)	European NAFLD registry	NASH					Obesity and T2D were more prevalent in NASH than in NAFL Negative correlations were reported between CLDQ scores and presence of obesity (*p* <0.001), T2D (*p* <0.001), and dyslipidaemia (*p* <0.01)
			54.9	54.9	54.9	54.9	54.9	
			NAFL					
			20.1	20.1	20.1	20.1	20.1	

**Quantitative studies (retrospective)**								

Alt *et al.* 2016^20^ Germany	Noninfectious CLD (150), including NASH (29)	Single-centre (January 2014 to June 2015)	69.0	27.6	48.3	69.0		Cirrhosis was reported in 34.5% of patients with NASH
**Qualitative studies**								

Cook *et al.* 2019^24^ UK and USA	NASH and fibrosis stages F1–F3 (16)	Physician referrals	75.0^c^	56.3	NR	NR	NR	Patients perceived other comorbidities (primarily T2D and obesity) to be more concerning than NASH They also felt that NASH is caused by overweight, and weight loss is key to resolving their disease
Doward *et al.* 2020^25^ USA	NASH: CE interviews (23) CD interviews (20)^f^	Single-centre (semistructured interviews)	CE					Other comorbidities included depression (CE 30.4%; CD 30.0%), asthma (CE 21.7%; CD 25.0%), and anxiety (CE: 17.4%; CD 20.0%)
			69.6	60.9	69.6	52.2	NR	
			CD					
			90.0	70.0	65.0	55.0	NR	
**Interventional studies**								

Armstrong *et al.* 2016^44^ UK	BC NASH (52)	Multicentre RCT (NCT01237119)	NR	32.7	55.8	30.8	5.8	
Younossi *et al.* 2018^37^ Canada and USA	Biopsy-proven NASH with stage F2/F3 fibrosis (72)	RCT (NCT02466516)	NR	70.8	76.4	NR	NR	Independent predictors of baseline PROs in patients with NASH included age, BMI, history of depression, anxiety, and hypertension
Younossi *et al.* 2019^39^ Global	Biopsy-proven NASH with advanced fibrosis^i^ who completed CLDQ-NASH (1,667)	Two RCTs (NCT03053050 and NCT03053063)	NR	69.4	NR	NR	NR	Other comorbidities (%) included cirrhosis (52.2) and psychiatric disorders (41.8); average BMI was 33.5 ± 6.6 Significantly impaired PRO scores by CLDQ-NASH were observed in patients with NASH and obesity (all domains *p* <0.001 except worry) and T2D (all *p* <0.01 except systemic)
Younossi *et al.* 2019^40^ Global	Biopsy-proven NASH with advanced fibrosis^i^ who completed PRO questionnaires^j^ Total: 1,667 Bridging fibrosis (F3): 797 CC (F4): 870	Two RCTs (NCT03053050 and NCT03053063)	F3					PRO scores were significantly lower in patients with NASH with T2D (*p* <0.01 for physical health-related domains and PCS of SF-36, EQ-5D utility, and 4 of 6 domains and the total score of CLDQ-NASH Other comorbidities (%) occurring in ≤25% of patients with F3/F4 fibrosis, respectively, included the following: • Blood and lymphatic system disorders: 26.8/15.1 • Endocrine disorders: 26.9/22.0 • GI disorders: 74.0/66.2 • Musculoskeletal disorders: 59.4/56.2 • Nervous system disorders: 39.7/38.4 • Psychiatric disorders: 44.1/39.1 • Respiratory disorders: 38.0/39.4 • Skin disorders: 27.5/30.2
			NR	77.2	NR	NR	15.5	
			F4					
			NR	70.3	NR	NR	19.2	
Younossi *et al.* 2020^41^ Global	Biopsy-proven NASH with advanced fibrosis^i^ Total: 1,699 Pruritus: 447^k^ No pruritus: 1,222^k^ Fatigue: 549^k^ No fatigue: 1,121^k^	Two RCTs (NCT03053050, NCT03053063)	With pruritus					Other comorbidities (%) occurring in ≤25% of patients with/without pruritus or with/without fatigue, respectively, included the following: • Blood and lymphatic system disorders: NR/NR/26.0/19.5 • Anxiety: 27.3/17.4/31.1/14.7 • Depression: 35.6/22.3/42.4/17.8 • GI disorders: 76.5/69.2/80.5/66.6 • Immune system disorders: 46.3/39.1/49.5/36.8 • Infections or infestations: 42.5/35.6/41.3/35.6 • Musculoskeletal disorders: 66.9/56.3/67.2/55.1 • Nervous system disorders: 50.8/36.7/53.2/34.3 • Respiratory disorders: 46.1/36.6/47.7/35.0 • Skin disorders: 39.4/26.3/31.9/28.7 • Vascular disorders: 75.2/69.5/72.3/70.4 • Eye disorders: 28.2/23.1/23.5/25.0 • Clinically significant itch^k^: 100/0/45.4/17.7
			NR	76.3	NR	NR	NR	
			No pruritus					
			NR	72.8	NR	NR	NR	
			With fatigue					
			NR	78.0	NR	NR	NR	
			No fatigue					
			NR	71.6	NR	NR	NR	
Younossi *et al.* 2021^42^ Global	Biopsy-proven NASH with advanced fibrosis^i^ Total: 2,154 Bridging fibrosis (F3): 1,021 CC (F4): 1,133	Four RCTs (NCT01672866, NCT01672879, NCT03053050, and NCT03053063)	F3					Other reported comorbidities (%) in F3/F4 patients, respectively, were as follows: • Clinically overt fatigue: 9.2/11.4 • GI disorders: 67.3/75.0 • Musculoskeletal and connective tissue disorders: 57.5/60.3 • Nervous system disorders: 39.3/41.6 • Psychiatric disorders: 39.7/44.7
			NR	70.2	NR	NR	NR	
			F4					
			NR	76.9	NR	NR	NR	

BC, biopsy-confirmed; CC, compensated cirrhosis; CD, cognitive debriefing; CE, concept elicitation; CLD, chronic liver disease; CLDQ, Chronic Liver Disease Questionnaire; CVD, cardiovascular disease; F1–4, fibrosis stages 1–4; GERD, gastro-oesophageal reflux disease; GfK, Growth from Knowledge; GGT, gamma-glutamyl transpeptidase; GI, gastrointestinal; HRQoL, health-related quality of life; MCS, mental component summary; NAFL, non-alcoholic fatty liver; NAFLD, non-alcoholic fatty liver disease; NASH, non-alcoholic steatohepatitis; NR, not reported; OR, odds ratio; PCS, physical component summary; PRO, patient-reported outcome; RCT, randomised controlled trial; SF-12, 12-item Short Form Health Survey; SF-36, 36-item Short Form Health Survey; T2D, type 2 diabetes.

a Obesity was defined as >30 or ≥30 kg/m^2^, unless otherwise specified.

b Representative sample of general population with varying health status.

c Obesity criteria not defined.

d With diabetes or pre-diabetes.

e Coronary artery disease.

f Eight participants took part in both groups.

g *p* <0.2 in univariate analyses was considered statistically significant.

h BMI >25 kg/m^2^.

i Bridging fibrosis (F3) or compensated cirrhosis (F4).

j SF-36, CLDQ-NASH, EQ-5D, and WPAI:SHP.

k Presence of fatigue and pruritus were indicated by a score of 4 or less on the respective items of the CLDQ-NASH (scale range 1–7).

Quality of life in patients with NASH deteriorated further when comorbidities such as T2D,^22^^,^^29^^,^^31^^,^[38], [39], [40] obesity,^31^^,^[37], [38], [39] hypertension,^29^^,^^37^ dyslipidaemia,^31^ depression/anxiety,^37^^,^^38^ and metabolic syndrome^29^ were present. For example, a database study of 1,338 patients with NASH and advanced fibrosis found greater impairment of physical health (SF-36 PCS scoring in the lowest quartile) in patients with higher BMI (odds ratio [OR] 1.058, 95% CI 1.036–0.081; *p* <0.0001), T2D (OR 1.54, 95% CI 1.11–2.12; *p* = 0.0093), and depression (OR 1.55, 95% CI 1.09–2.19; *p* = 0.014) than those with lower BMI, no T2D, and no depression.^38^ Another study found that patients tended to perceive other comorbidities (primarily T2D and obesity) as more concerning than NASH.^24^

### Quality of included studies

The 17 quantitative studies included in the review were assessed using the EPHPP tool. Of these, 14 studies were rated as ‘moderate’ and 3 studies as ‘weak’ (Table S4). Because most of the studies were observational in nature, the overall global rating of the studies was impacted by missing data across publications regarding withdrawals/drop-outs. In addition, all 17 studies received a ‘weak’ rating for their information on confounders. Quality assessment of the RCTs is provided in Table S5. Three RCTs scored well on all 7 components.^39^^,^^43^^,^^44^ Two did not provide clear descriptions for 2 or 3 categories,^32^^,^^37^ and 1 study did not provide sufficient information to judge its quality in 6 of 7 categories.^42^ The most common category with quality concerns was adequate concealment of treatment allocation, which was unclear for 3 studies.^32^^,^^37^^,^^42^

## Discussion

The findings of this study provide a detailed picture of the humanistic burden of NASH. Utilising 25 publications describing 23 unique studies, we show that there is a substantial impact of NASH/NAFLD on patients’ HRQoL, especially in terms of physical functioning and fatigue. This high burden is associated with a considerable deterioration of physical and mental health that becomes more evident as NASH progresses.

Nineteen studies reported data on populations with NASH exclusively, and 4 studies reported data on subgroups with NASH within populations with NASH/NAFLD. Although a broad search strategy and screening criteria were used, the included studies focussed on populations with NASH and NASH/NAFLD to answer the specific research question around the humanistic burden of NASH. The data related to patients with NASH were heterogenous with regard to NASH diagnostic methods, population, outcomes, follow-up time, and measures of HRQoL/utility. There was a mixed use of liver- or NASH-specific and generic HRQoL instruments among the studies, with some studies using both instruments to assess validity.

The generic SF-36 and liver-specific CLDQ instruments were used most frequently across the studies. Although criticisms exist regarding the use of non-disease-specific instruments for assessment of individuals with chronic diseases, SF-36 and CLDQ have been shown to be reliable in populations with NASH, and the studies identified in this review have provided useful information. In fact, the reliability and validity of the CLDQ when compared with those of SF-36 have been evaluated specifically in patients with NASH.^22^ This study showed significant correlations between SF-36 and CLDQ,^22^ and another study confirmed the correlation between SF-36 and most domains of CLDQ-NASH.^39^ In the latter study, only the NASH-specific domains of ‘abdominal’ and ‘worry’ did not correlate with SF-36 domains. The authors note that CLDQ-NASH shares 29 of its 36 items with the original CLDQ, and the additional items that allow for more NASH-specific assessment will likely add to its reliability.^39^

One qualitative study was undertaken to inform the development of a new NASH-specific instrument.^25^ Doward *et al.*^25^ used concept elicitation interviews and cognitive debriefing to develop and validate the pilot version of NASH-CHECK, a 31-item PRO tool. NASH-CHECK is not validated to the same extent as CLDQ-NASH, the most frequently used disease-specific tool, and full data for the psychometric characteristics of NASH-CHECK are not yet available. Once fully validated, NASH-CHECK may become relevant and acceptable to patients and clinicians. Primary endpoints in NASH clinical trials typically include measures of histological disease manifestations, but these do not capture the humanistic impact of NASH. Incorporation of validated PROs that capture the patient perspective can add value to clinical trial results and complement clinical endpoints.^25^^,^^45^ PRO tools can also be of value to the field, and their inclusion into clinical trials could support their future validation.

Despite the evidence supporting the clinical burden of NASH, appreciation for the impact of NASH on patients’ quality of life is quite limited. In fact, a frequent perception exists that NASH is an asymptomatic condition, particularly in its early stages.^25^^,^^31^^,^^38^ The quantitative studies included in this systematic review dispel this misconception, showing that NASH is associated with significantly reduced HRQoL compared with the general population.^21^^,^^22^^,^^27^^,^^37^^,^^38^^,^^40^ Some studies indicated that NASH predominately impacts patients’ physical function,^37^^,^^38^ although most showed significant impairments in HRQoL across the mental and physical domains of the SF-36 and CLDQ, as well as SF-6D and EQ-5D utility scores and WPAI scores.[21], [22], [23]^,^^27^^,^^40^ NASH was also associated with worse HRQoL scores than those of matched participants with T2D (SF-36 MCS and health utility) and chronic hepatitis C (physical SF-36 domains and fatigue on CLDQ),^21^^,^^38^ further supporting the significant burden of illness with NASH.

The most prevalent symptoms associated with NASH were fatigue, abdominal pain, anxiety/depression, cognition problems, and poor sleep quality.^21^^,^[23], [24], [25]^,^^28^^,^^41^ Symptoms of NASH were associated with worse HRQoL scores across several PROs, including SF-36, CLDQ, CLDQ-NASH, WPAI:SHP, and health utility instruments,^28^^,^^41^ and were found to be detrimental to many aspects of patients’ daily lives, including their ability to work and perform daily living tasks, and the quality of both family and intimate relationships.^24^^,^^25^ Despite these varied symptoms and their consequences, 2 studies indicated that some patients do not attribute their symptoms to NASH, often attributing them to other comorbid conditions or feeling uncertain of their origin,^23^^,^^24^ which may contribute to the misperception that chronic liver diseases such as NASH are asymptomatic. One study reported that NASH diagnoses were incidental in most of their patient population, largely because patients failed to associate their signs or symptoms with their liver condition.^24^

It was shown that patients demonstrate a general lack of understanding of their disease, feel that other comorbid conditions are more concerning than NASH itself, and do not seem familiar with consequences of NASH in the long term.^24^ Further, they felt a lack of adequate educational support from their physicians. Similar findings were reported in a recent systematic review that assessed quality of life in over 37,000 patients with chronic liver disease (including NASH), in which patients call for better education and information to understand and manage their disease.^46^ Education of patients with NASH on their liver condition, diagnosis, and the progressive nature of the disease could lead to better disease management and improved patient outcomes.

The systematic review from Grønkjær and Lauridsen^46^ also found that patients with chronic liver diseases, including NASH, have inadequate support owing to limited knowledge of liver diseases in the general population, resulting in loneliness and social isolation. This adds weight to the finding that lower perceived social support from friends and relatives is associated with less activity and vitality, greater anxiety, and more denial among patients with NASH.^27^ The importance of support networks in patient health has been demonstrated in a variety of conditions, including cancer, heart failure, multiple sclerosis, and liver transplant candidates.[47], [48], [49], [50] Promotion of active coping skills and education of patients and their families on the importance of effective social networks and/or formal support sources may improve the overall health and well-being of patients with NASH.

Unsurprisingly, patients with NASH had significantly more functional impairment than those with simple steatosis,^26^^,^^27^^,^^31^ and patients with more progressive NASH generally exhibited worse HRQoL scores.^27^^,^^31^^,^^35^^,^^39^^,^^40^^,^^42^ HRQoL by fibrosis stage was assessed in several studies. One showed worsening HRQoL with progression of fibrosis,^35^ and another reported a trend toward worse HRQoL in patients with advanced fibrosis and compensated cirrhosis (F3/F4 *vs.* F0–2).^31^ In contrast, a third study found no difference in HRQoL scores between patients with F2 fibrosis and those with F3 fibrosis.^37^

Worse HRQoL was also evident in patients with more severe hepatic steatosis, ballooning, and lobular inflammation, the latter of which was a surprising finding since greater lobular inflammation does not independently correlate with overall or liver-specific mortality.^31^ The authors note that improvement of steatosis, in particular lobular inflammation, may result in a measurable improvement in HRQoL, even independently of fibrosis improvement. This is an interesting finding as many economic evaluations tend to focus on change in fibrosis stage alone, so the value of changes in histological parameters such as steatosis, ballooning, and lobular inflammation is always captured.^51^ As mentioned above, the recommended primary endpoints for clinical trials in patients with pre-cirrhotic NASH do include ‘resolution of steatohepatitis and no worsening of fibrosis’ and ‘≥2-point reduction in NAS with ≥1-point reduction in either lobular ballooning or hepatocellular ballooning and no worsening of fibrosis’,[52], [53], [54] but the absence of data linking histological parameters to long-term outcomes may prove a barrier to use in some studies. Indeed, fibrosis currently remains the only independent predictor of long-term outcomes such as mortality in NAFLD.[55], [56], [57], [58]

Change in non-invasive markers of NASH/fibrosis severity were shown to correlate with change in HRQoL in 2 interventional studies.^37^^,^^42^ Similar findings were reported in 2 recent studies in the literature that examined the impact of treatment with obeticholic acid or resmetirom on quality of life.^59^^,^^60^ Interim results from the REGENERATE study, which is assessing the safety and efficacy of obeticholic acid in patients with NASH, report greater improvement in some PRO domains in patients experiencing fibrosis improvement (total CLDQ-NASH score and fatigue and worry scores), decreased NAS (emotional and worry scores), or NASH resolution without worsening of fibrosis (worry score).^59^ However, treatment with obeticholic acid itself was not associated with any HRQoL improvements *vs*. placebo after 18 months of treatment. In a phase II placebo-controlled study, resmetirom-treated patients experienced improvement of bodily pain and SF-6D scores at Week 12 and PCS improvement up to Week 36, with no improvement seen in the placebo group.^60^ Adjusted analyses found that improvement in hepatic fat fraction was independently associated with greater HRQoL improvements, and improvement in NAS and fibrosis on serial liver biopsy was also linked to improvement in HRQoL components. Only 1 placebo-controlled study, the PIVENS study, was included in this systematic review, and in a surprising contrast to the aforementioned studies, no difference in the change in HRQoL was seen between placebo and vitamin E or pioglitazone, despite improvements in NASH histological features and NAS with both active treatments.^43^

Understanding the impact of cirrhosis on HRQoL is important because there is a growing prevalence of NASH-related liver failure, as highlighted by US registry data for liver transplantation.^11^^,^^12^ Although 1 single-centre study found no significant difference in function between patients with pre-cirrhotic and cirrhotic NAFLD,^26^ the results overall suggest that the HRQoL burden is likely to be higher in patients with NASH who have progressed to cirrhosis, HCC, or liver failure. Similar findings were reported in a recent study in the literature, which showed that patients with NASH–cirrhosis have lower HRQoL and poorer physical health than patients with non-cirrhotic NASH.^61^

Despite large variation in reported comorbidity rates across the studies, rates of obesity, diabetes, hypertension, hyperlipidaemia/dyslipidaemia, and CVD were generally high,[20], [21], [22], [23], [24], [25]^,^^28^^,^^29^^,^^31^^,^^33^^,^[35], [36], [37], [38], [39]^,^^44^ indicating a high disease and clinical management burden. Several studies reported worse HRQoL in patients with comorbidities than in those without comorbidities (such as T2D, obesity, and metabolic syndrome),^22^^,^^29^^,^^31^^,^[38], [39], [40] although it is unclear what proportion of the NASH symptom burden should be attributed to the disease itself, or to patients’ comorbidities, particularly in light of the weak rating for control of confounders noted in all quantitative studies. The impact of comorbidities on HRQoL is of relevance given the significant increase in obesity-related conditions across the world in recent decades and the accompanying increase in weight-related mortality.^62^ Unfortunately, 1 study noted that patients can interpret physician recommendations for weight loss as relatively unimportant in relation to NASH, as this is routine guidance given regardless of their medical condition,^23^ highlighting again the need for improved education on NASH, its management, disease progression, and impact of comorbidities, for both patients and healthcare providers.

Finally, it is important to note that the histologic endpoint of NASH may be increasingly replaced by NITs in future studies and practice. In 1 study, in which patients with NASH with liver biopsy and NITs were assessed using CLDQ-NASH and SF-36,^42^ both histologic stage and NIT scores indicating high risk for advanced fibrosis were associated with poor PRO scores.^42^ This study suggests the importance of associating PROs with NITs assessing hepatic fibrosis as well as with histologic endpoints.

### Study and data limitations

This review was conducted as per systematic literature review guidelines with a robust methodology; however, there are certain inherent limitations with most literature reviews. This systematic literature review was limited to English language and restricted to those published since January 2010 to examine the most recent evidence. Most of the studies were conducted in Europe or North America, and almost half were cross-sectional in nature. The studies also included heterogenous patient populations and used varying definitions to diagnose NASH, limiting the comparability of the data. In addition, few studies that compared HRQoL in populations with NASH with that in other populations included matched populations adjusted for factors such as income, education, alcohol intake, and comorbidities.

Several studies using NASH-specific PRO instruments are underway, although studies using instruments other than CLDQ-NASH are limited, and further research utilising disease-specific measures is justified to fully understand the humanistic burden of NASH. Although limited evidence is available, significant correlations between generic and liver disease-specific PRO instruments were reported, with both showing the high humanistic burden of NASH and greater impact among patients with more severe disease.

The quality assessment of the quantitative studies showed that all evaluable studies were rated as ‘moderate’ (n = 14) or ‘weak’ (n = 3), highlighting the need for more robust evidence on the HRQoL impact of NASH. Ratings are primarily attributable to the observational nature of most studies. Owing to the varied study designs, there is a possibility for comorbidities to confound HRQoL results, and notably, all quantitative studies scored weakly for control of confounders. This is particularly relevant given the high rates of comorbid conditions throughout the included studies, and the close links between NASH/NAFLD and T2D, obesity, and cardiovascular outcomes. As such, it is recommended that future studies consider comorbidities as important factors and use designs and methods to better control the potential of confounding caused by comorbidities and to promote our knowledge on the relationship between comorbidities and HRQoL in patients with NASH/NAFLD.^50^

### Conclusions

This systematic review identified a significant and often unrecognised HRQoL burden among individuals with NASH. This burden increases as the disease progresses, adversely impacting patients’ family relationships and their ability to work and perform activities of daily living. These findings also highlighted a need for better quality studies examining the humanistic burden of NASH. In particular, HRQoL studies need to adjust for the presence of comorbidities that may influence outcomes.

## Financial support

This study was funded by 10.13039/501100004191Novo Nordisk A/S, Søborg, Denmark. Novo Nordisk A/S was responsible for the study concept and design, data collection, and the decision to submit this article for publication. With the input of all authors, Novo Nordisk A/S was involved in the analysis and interpretation of data and writing of this report.

## Authors' contributions

Provided the study concept and design: PJ. Performed the data extraction: JF, PA, SN. Contributed to the analysis and interpretation of the data and contributed to the drafting of the paper, critically revised the paper for intellectual content, were involved in the final approval of the version to be published and agreed to be accountable for all aspects of the work: all authors. Overall guarantor: MA.

## Data availability statement

Data sharing is not applicable to this article as no datasets were generated or analysed during the current study.

## Conflicts of interest

IS, JF, PJ, and MA are employees of Novo Nordisk A/S or Novo Nordisk Denmark A/S. PJ, IS, and JF are also shareholders of Novo Nordisk A/S. ZY has received research funding and/or serves as a consultant to Abbott, AbbVie, Bristol Myers Squibb, Gilead Sciences, Intercept, Madrigal, Merck, Novartis, Novo Nordisk, Quest, Siemens, Terns, and Viking. The SLR was commissioned to DRG Abacus (Clarivate) by Novo Nordisk, and PA and SN are employees of DRG Abacus (Clarivate).

Please refer to the accompanying ICMJE disclosure forms for further details.
