# Supplementary material for: The burden of non-alcoholic steatohepatitis: A systematic review of health-related quality of life and patient-reported outcomes
Source: JHEP Rep. 2022 Jun 15;4(9):100525. doi: 10.1016/j.jhepr.2022.100525 (PMC9418497; doi:10.1016/j.jhepr.2022.100525)
Supplement: Multimedia component 1 [file mmc1.pdf]

The burden of non-alcoholic steatohepatitis: A systematic review of health-related quality of life and patient-reported outcomes

Zobair Younossi, Priya Aggarwal, Ichhya Shrestha, João Fernandes, Pierre Johansen, Margarida Augusto, Sunita Nair

Table of contents

Supplementary methods 2

Supplementary results.. 3

Table S1 5

Table S2. 12

Table S3. 14

Table S4 20

Table S5. 21

Table S6 22

Table S7. 36

Supplementary references 38

Supplementary methods

Literature searches included terms for free text and Medical Subject Heading for Medline and the Cochrane Library, or Emtree terms for EMBASE, related to non-alcoholic steatohepatitis or non-alcoholic fatty liver disease, and combined with terms related to outcomes of interest.

Relevant conference proceedings from 2017 to 2021 were hand-searched as a supplementary measure to identify further relevant studies that were not captured in the electronic database searches. These included: European Association for the Study of Diabetes, American Diabetes Association Scientific Sessions, American Association for the Study of Liver Diseases, the International Liver Congress, European Association for the Study of Liver Diseases, the Asian Pacific Association for the Study of the Liver and the International Society for Pharmacoeconomics and Outcomes Research.

Conference abstracts were identified and screened but data were not extracted as it was anticipated that the large number of full-text publications would provide an adequate evidence base of relevant information.

Submission documents from the National Institute for Health and Care Excellence, Scottish Medicine Consortium, Canadian Agency for Drugs and Technologies in Health, Pharmaceutical Benefits Advisory Committee and Haute Autorité de Santé were also reviewed for relevant data.

Supplementary results

Disease focus and instruments used

Most studies reported NASH population data exclusively [1-19], while four studies described results in NASH subgroups within NASH/NAFLD populations [20-23]. The studies included heterogenous patient populations with varying definitions used to diagnose NASH. The most used technique for confirming NASH diagnosis was biopsy, potentially reflecting a bias for inclusion of patients with biopsy-proven disease in clinical trials. Other methods (which can only be used for diagnosis of NAFLD) included transient elastography [1, 5], ultrasound [5, 6, 9, 11, 13] and self-reported physician diagnosis [3]. A detailed description of all patient characteristics across all studies is reported in **Table S6**.

Most studies measured HRQoL using instruments and PROs. These included 15 quantitative [1, 3-5, 8-10, 12-14, 17, 20-23] and six interventional studies. The latter comprised three RCTs [2, 11, 15], two post-hoc analyses of one [16] and four [19] RCTs, and one study based on patients from two RCTs that was reported over three publications (one PRO validation and two post-hoc analyses) [18, 24, 25].

The generic Short Form-36 (SF-36) instrument and the liver-specific Chronic Liver Disease Questionnaire (CLDQ) emerged as the most frequent choices to assess quality of life, being used in nine and six studies, respectively (**Table S7**). Other instruments included the Short Form-6 Dimension (SF-D6), EuroQol-5D (EQ-5D), Patient-Reported Outcome Measurement Information System Health Assessment Questionnaire (PHAQ), School Physical Activity and Nutrition, Work Productivity and Activity Impairment: Specific Health Problem (WPAI:SHP), Hospital Anxiety and Depression Scale (HADS), Beck Depression Inventory-II (BDI-II) and the disease-specific CLDQ-NASH (also referred to as CLDQ-NAFLD in some studies). One publication also reported the development of NASH-CHECK, a new NASH-specific PRO measure to assess quality of life [7].

Two qualitative studies used different assessment tools to provide useful insights on symptoms experienced by NASH patients and their impact on patients: one used an online qualitative market research tool (online bulletin board) followed by in-depth telephone interviews [6], and the other used semi-structured, face-to-face, concept elicitation interviews [7].

Validity of liver- and NASH-specific instruments

Two studies assessed the level of correlation between the generic SF-36 and liver- or disease-specific measures [4, 24]. Chawla and colleagues (2016) [4] evaluated HRQoL using SF-36 and CLDQ in 79 participants with NASH, reporting highly significant correlations between overall CLDQ score and SF-36 physical component summary (PCS; $r = 0.82$, $p<0.0001$) and mental component summary (MCS; $r = 0.67$, $p<0.0001$) scores, and similar degrees of correlation between relevant subscales. Highly significant correlations were also observed between subscales of the NASH-specific CLDQ-NASH with SF-36 domains in 1667 patients with biopsy-proven NASH [24]. The most highly correlated domains were activity of CLDQ-NASH with SF-36 physical functioning, emotional of CLDQ-NASH with SF-36 mental health, fatigue of CLDQ-NASH with SF-36 vitality and systemic symptoms of CLDQ-NASH with SF-36 bodily pain (all rho $\geqslant 0.70$, $p<0.0001$). In contrast, the CLDQ-NASH domains of abdominal and worry did not correlate with SF-36 domains (all rho $\leqslant 0.50$), reflecting the disease-specific nature of these domains.

Supplementary tables

Table S1. Search strategies for identification of publications for systematic review.

#	Searches - Embase <1974 to 2020 Week 53>	Results	
		Primary SLR	SLR update
1	exp nonalcoholic fatty liver/	38 036	46 829
2	(non-alcoholic steatohepatitis or nonalcoholic steatohepatitis or NASH or NAFLD).mp.	37 523	44 179
3	exp nonalcoholic steatohepatitis/	38 036	4749
4	1 or 2 or 3	48 053	55 905
5	(EuroQOL 5-Dimension or Euroqol 5D or EQ-5D or EQ5D or Euroqol).mp.	19 749	22 333
6	(Health utilities index or HUI).mp.	3271	3380
7	(time trade off or time trade-off or ("TTO" adj2 "time trade")).mp.	1728	1832
8	(short form 6D or short-form 6D).mp.	317	332
9	(standard gamble or ("SG" adj2 "standard gamble")).mp.	1103	1137
10	(15D or 16D or 17D).mp.	4009	4177
11	exp short form 12/ or exp short form 20/ or exp short form 36/	33 909	37 763
12	("quality of well-being index" or "quality of wellbeing index" or "quality of well being index").mp.	24	26
13	(medical outcome adj1 (survey or stud*)).mp.	1114	1165
14	(life year* lost or years of life lost or (years adj2 with disability)).mp.	2500	2809
15	(quality adjusted life year* or QALY*).mp.	31 822	34 436
16	(QoL or HRQoL or HRQL).mp.	98 630	108 318
17	exp "quality of life"/	473 522	513 942
18	(health related quality of life or health-related quality of life).mp.	62 363	68 766
19	((quality of life or QoL) adj10 (question$ or instrument or scale$1or score$1)).mp.	107 606	120 371

20	health state$.mp.	10 948	11 769
21	utilit*.mp.	285 241	307 362
22	Patient Preference/ or preference.mp.	151 108	159 012
23	(map$ or regression).mp.	1 753 342	1 891 991
24	exp health status/	230 820	247 220
25	health survey/	189 249	195 748
26	exp daily life activity/	86 482	92 091
27	("Activities of Daily Living" or "IADL").mp.	38 999	42 413
28	Psychometrics.mp. or exp psychometry/	90 209	94 601
29	("health year equivalent" or "HYE").mp.	115	122
30	or/5-29	3 030 518	3 253 255
31	4 and 30	6795	8242
32	exp animal/ not (exp human/ and exp animal/)	4 574 286	4 367 840
33	exp note/ or exp editorial/ or exp letter/ or exp case report/ or exp Case study/ or exp Abstract report/ or (review or letter or editorial or note).pt.	7 315 375	7 555 031
34	or/32-33	11 651 963	11 696 067
35	31 not 34	5674	6960
36	limit 35 to conference abstracts	2836	3582
37	35 not 36	2838	3378
38	limit 37 to english language	2707	3235
39	limit 38 to yr="2010 -Current"	2380	–
40	limit 36 to yr="2017 -Current"	1179	–
41	39 or 40	3559	–
42	(Feb* 2020 or Mar* 2020 or Apr* 2020 or May* 2020 or Jun* 2020 or Jul* 2020 or Aug* 2020 or Sep* 2020 or Oct* 2020 or Nov* 2020 or Dec* 2020).dp.	–	445 544
43	38 and 39	–	158
44	36 and 39	–	446

45	limit 38 to yr="2021 -Current"	–	10
46	limit 36 to yr="2021 -Current"	–	0
47	40 or 41 or 42 or 43	–	614
**#**	**Searches - Ovid MEDLINE(R) and Epub Ahead of Print, In-Process & Other Non-Indexed Citations and Daily <1946 to January 04, 2021>**	**Results**	
		**Primary SLR**	**SLR update**
1	exp nonalcoholic fatty liver/	11 275	13 331
2	(non-alcoholic steatohepatitis or nonalcoholic steatohepatitis or NASH or NAFLD).mp.	20 552	23 950
3	exp nonalcoholic steatohepatitis/	11 275	13 331
4	1 or 2 or 3	22 914	26 707
5	(EuroQOL 5-Dimension or Euroqol 5D or EQ-5D or EQ5D or Euroqol).mp.	10 415	11 898
6	(Health utilities index or HUI).mp.	1624	1764
7	(time trade off or time trade-off or ("TTO" adj2 "time trade")).mp.	1159	1214
8	(short form 6D or short-form 6D).mp.	136	146
9	(standard gamble or ("SG" adj2 "standard gamble")).mp.	838	861
10	(15D or 16D or 17D).mp.	2778	2888
11	((short-form adj ("12" or "16" or "20" or "36")) or SF-12 or SF12 or SF-16 or SF16 or SF-20 or SF20 or SF-36 or SF36).mp.	30 864	32 912
12	("quality of well-being index" or "quality of wellbeing index" or "quality of well being index").mp.	18	18
13	(medical outcome adj1 (survey or stud*)).mp.	819	850
14	(life year* lost or years of life lost or (years adj2 with disability)).mp.	1960	2218
15	(quality adjusted life year* or QALY*).mp.	18 602	20 211
16	(QoL or HRQoL or HRQL).mp.	54 374	59 912
17	exp "quality of life"/	188 046	202 132
18	(health related quality of life or health-related quality of life).mp.	42 704	46 943

19	((quality of life or QoL) adj10 (question$ or instrument or scale$1or score$1)).mp.	44 381	48 285
20	health state$.mp.	6275	6783
21	utilit*.mp.	202 581	219 400
22	Patient Preference/ or preference.mp.	108 896	116 317
23	(map$ or regression).mp.	1 447 867	1 559 133
24	exp health status/	327 514	350 494
25	health survey/	61 271	63 568
26	exp "Activities of Daily Living"/	99 640	104 508
27	("Activities of Daily Living" or "IADL").mp.	76 210	79 689
28	Psychometrics.mp.	75 226	79 006
29	("health year equivalent" or "HYE").mp.	51	55
30	or/5-29	2 225 068	2 388 492
31	4 and 30	2934	3465
32	exp animal/ not (exp human/ and exp animal/)	4 672 546	4 772 383
33	exp note/ or exp editorial/ or exp letter/ or exp case report/ or exp Case study/ or (review or letter or editorial or note).pt.	5 910 216	6 190 814
34	or/32-33	10 354 851	10 730 013
35	31 not 34	2486	2950
36	limit 35 to english language	2405	2860
37	limit 36 to yr="2010 -Current"	2152	–
38	limit 36 to dt=20200201-20201231	–	473
39	limit 36 to yr="2021 -Current"	–	9
40	or/37-38	–	473
**#**	**Cochrane Central Register of Controlled Trials January 2020; Cochrane Database of Systematic Reviews 2005 to January 4, 2021; Database of Abstracts of Reviews of Effects 1st Quarter 2016; Health Technology Assessment**	**Results**	
		**Primary SLR**	**SLR update**

	**4th Quarter 2016; NHS Economic Evaluation Database 1st Quarter 2016**		
1	exp nonalcoholic fatty liver/	0	0
2	(non-alcoholic steatohepatitis or nonalcoholic steatohepatitis or NASH or NAFLD).mp.	2620	3003
3	exp nonalcoholic steatohepatitis/	0	0
4	1 or 2 or 3	2620	3003
5	(EuroQOL 5-Dimension or Euroqol 5D or EQ-5D or EQ5D or Euroqol).mp.	9658	10 195
6	(Health utilities index or HUI).mp.	593	508
7	(time trade off or time trade-off or ("TTO" adj2 "time trade")).mp.	545	194
8	(short form 6D or short-form 6D).mp.	57	52
9	(standard gamble or ("SG" adj2 "standard gamble")).mp.	292	94
10	(15D or 16D or 17D).mp.	333	331
11	((short-form adj ("12" or "16" or "20" or "36")) or SF-12 or SF12 or SF-16 or SF16 or SF-20 or SF20 or SF-36 or SF36).mp.	17 117	18 485
12	("quality of well-being index" or "quality of wellbeing index" or "quality of well being index").mp.	11	3
13	(medical outcome adj1 (survey or stud*)).mp.	258	261
14	(life year* lost or years of life lost or (years adj2 with disability)).mp.	166	104
15	(quality adjusted life year* or QALY*).mp.	10 478	5941
16	(QoL or HRQoL or HRQL).mp.	24 023	26 224
17	exp "quality of life"/	23 682	23 872
18	(health related quality of life or health-related quality of life).mp.	17 733	18 878
19	((quality of life or QoL) adj10 (question$ or instrument or scale$1or score$1)).mp.	27 851	31 101
20	health state$.mp.	2622	1316
21	utilit*.mp.	21 549	17 782

22	Patient Preference/ or preference.mp.	11 795	12 224
23	(map$ or regression).mp.	82 381	86 877
24	exp health status/	7590	7665
25	health survey/	868	897
26	exp "Activities of Daily Living"/	5277	5397
27	("Activities of Daily Living" or "IADL").mp.	11 492	11 876
28	Psychometrics.mp.	3073	3108
29	("health year equivalent" or "HYE").mp.	15	14
30	or/5-29	196 874	203 398
31	4 and 30	348	410
32	exp animal/ not (exp human/ and exp animal/)	46	12
33	exp note/ or exp editorial/ or exp letter/ or exp case report/ or exp Case study/ or (review or letter or editorial or note).pt.	10 978	11 278
34	or/32-33	11 024	11 290
35	31 not 34	348	410
36	limit 35 to english language	288	342
37	limit 36 to yr="2010 -Current"	272	–
38	limit 36 to yr="2021 -Current"	–	31
**#**	**Searches - PsycINFO <1806 to January Week 1 2021>**	**Results**	
		**Primary SLR**	**SLR update**
1	(non-alcoholic steatohepatitis or nonalcoholic steatohepatitis or NASH or NAFLD).mp.	1593	1107
2	steatohepatitis.mp.	126	107
3	NAFLD.mp.	103	109
4	NASH.mp.	1483	995
5	(non-alcoholic or "non alcoholic").mp.	568	326
6	or/1-5	2126	1393
7	limit 5 to yr="2010 -Current"	258	

8	(Nash and (bargain* or equilibr* or strateg* or policy or game or network* or welfar*)).mp.	1097	763
9	7 not 8	258	–
10	((non-alcoholic or "non alcoholic") and beverage?).mp.	123	104
11	9 not 10	178	–
12	limit 11 to human	145	–
13	6 not (7 or 8)	–	526
14	limit 9 to human	–	424
15	limit 10 to up=20200201-20201123	–	13
16	limit 10 to yr="2021 -Current"	–	3
17	or/11-12	–	15

SLR, systematic literature review.

Table S2. Criteria for eligibility for systematic review.

Criteria	Include	Exclude
Population	• Patients with NASH*<br>• Patients with NAFLD having subgroup data separately for NASH*	• Patients without NAFLD or NASH<br>• Patients with NAFLD without having subgroup analysis for patients with NASH
Interventions/ comparators	• No restriction	• No restriction
Outcomes	• HRQoL or PROs, including:<br>o NASH-CHECK<br>o SF-36<br>o SF-6D<br>o CLDQ<br>o CLDQ-NASH<br>o WPAI:SHP<br>o Outcomes related to ADL, including: fatigue, pain, functional impairment, or activity limitations	• Outcomes not reporting on HRQoL or PROs
Study design/setting	• No restriction	• Case studies, and case-control studies, editorials, letters, commentaries<br>• Reviews<br>• Systematic reviews[†]
Language of publication	• English language publications	• Studies published in languages other than English
Date of publication	• Full-text publications: 2010−January 2021	• Full-text publications: published before 2010

Criteria	Include	Exclude
	• Conference proceedings: 2017–2021[‡]	• Conference proceedings: published before 2017

ADL, activities of daily living; CLDQ, Chronic Liver Disease Questionnaire; HRQoL, health-related quality of life; NAFLD, non-alcoholic fatty liver disease; NASH, non-alcoholic steatohepatitis; PROs, patient-reported outcomes; SF-36, Short Form-36; SF-6D, Short Form-6D; SLR, systematic literature review; WPAI:SHP, Work Productivity and Activity Impairment: Specific Health Problem.

*As defined by individual study inclusion criteria; [†]SLRs were included in the supplementary searches (reference lists screening); [‡]During the 2021 update, conference abstracts of the last year were included but not extracted for analysis. During title/abstract screening, conference abstracts identified from the electronic database with NAFLD data were also considered prior to the decision to focus on NASH.

Table S3. Overview of HRQoL and PRO instruments.

**Instrument/ scale**	**No. items**	**Response format**	**Recall period**	**Domains (items per domain)**	**Possible range**	**Reference**
Generic measures						
Athens Insomnia Scale (AIS)	8	4-option Likert scale	1 month	Sleep induction (1)<br>Awakenings during the night (1)<br>Final awakening (1)<br>Total sleep duration (1)<br>Sleep quality (1)<br>Wellbeing during the day (1)<br>Functioning capacity during the day (1)<br>Sleepiness during the day (1)	0–24<br>0 denotes absence of any sleep-related problem<br>24 denotes most severe degree of insomnia	Soldatos et al 2000 [26]
Beck Depression Inventory®-II (BDI®-II)	21	4-option Likert scale*	2 weeks	N/A	0–63<br>0–13 indicates minimal depression<br>14–19 indicates mild depression<br>20–28 indicates moderate depression	Beck et al 1996 [27]

Instrument/ scale	No. items	Response format	Recall period	Domains (items per domain)	Possible range	Reference
					29–63 indicates severe depression	
Frequency scale for the symptoms of GERD (FFSG)	12	5-option Likert scale	N/A	N/A	0–48<br>Scores ≥8 indicate GERD	Kusano et al 2004 [28]
Hospital Anxiety and Depression Scale (HADS)	14	4-option Likert scale	1 week	Depression (7)<br>Anxiety (7)	0–21 per domain<br>Total score 8–10 indicates borderline abnormal case<br>Total score 11–21 indicates abnormal case	Zigmond and Snaith 1983 [29]<br>Stern 2014 [30]
Patient-Reported Outcome Measurement Information System (PROMIS) Health Assessment Questionnaire (PROMIS-HAQ)	20	5-option Likert scale	Present	Dressing<br>Arising<br>Eating<br>Walking<br>Hygiene<br>Reach<br>Grip<br>Activity	0–100<br>Higher scores indicate greater functional impairment	Fries et al 2009 [31]<br>Elliott et al 2013 [20]

Instrument/ scale	No. items	Response format	Recall period	Domains (items per domain)	Possible range	Reference
School Physical Activity and Nutrition (SPAN)[†]	Age 9–10: 74<br>Age 13–17: 108	Mixed	24 hours	N/A	N/A	Michael & Susan Dell Center for Healthy Living (n.d.) [32]
12-item Short-Form Health Survey (SF-12)	12	Mixed, ranging from dichotomous (yes/no) to 6-option Likert scale	Now to 4 weeks	**Physical component:**<br>Physical functioning (2)<br>Role physical (2)<br>Bodily pain (1)<br>General health (1)<br>**Mental component:**<br>Vitality (1)<br>Social functioning (1)<br>Role emotional (2)<br>Mental health (2)	0–100<br>0 denotes poor health<br>100 denotes excellent health	Ware et al 1996 [33]
36-item Short-Form Health Survey (SF-36)	36	Mixed, ranging from dichotomous (yes/no) to 6-option Likert scale	Now to 4 weeks	**Physical component:**<br>Physical functioning (10)<br>Role physical (4)<br>Bodily pain (2)<br>General health (5)	0–100<br>0 denotes poor health<br>100 denotes excellent health<br><br>PCS: 8–73<br>MCS: 10–74	Maruish 2011 [34]<br>RAND Health Care (n.d.) [35]

Instrument/ scale	No. items	Response format	Recall period	Domains (items per domain)	Possible range	Reference
				**Mental component:**<br>Vitality (4)<br>Social functioning (2)<br>Role emotional (3)<br>Mental health (5)<br>**Other:**<br>Health transition (1)		
Work Productivity and Activity Impairment Questionnaire (WPAI)‡	5	Mixed	1 week	**Employed persons:**<br>Absenteeism<br>Presenteeism<br>**All persons:**<br>Activity impairment	0–100<br>0 denotes less impairment and more productivity<br>100 denotes more impairment and less productivity	Reilly et al 1993 [36]<br>Reilly Associates (2019) [37]
Multi-attribute and preference-based utility measures						
Visual Analogue Scale (VAS)	1	Continuous scale	Today	N/A	0–100<br>0 denotes worst imaginable health<br>100 denotes best imaginable health	

Instrument/ scale	No. items	Response format	Recall period	Domains (items per domain)	Possible range	Reference
Standard Gamble (SG)	N/A	Recursive method	N/A	N/A	0–1<br>0 denotes death<br>100 denotes ideal health	
EuroQol-5D (EQ-5D)	5-item descriptive system plus EQ-VAS	**Descriptive:** 3-option (EQ-5D) or 5-option (EQ-5D-5 level) Likert scales<br>**EQ-VAS:** continuous scale	Today	**Descriptive system:**<br>Mobility (1)<br>Self-care (1)<br>Usual activities (1)<br>Pain/discomfort (1)<br>Anxiety/depression (1)<br><br>**EQ-VAS:** N/A	0–1<br>0 denotes death<br>100 denotes ideal health	EuroQol (n.d.) [38]
Short Form-6 Dimension (SF-6D)	6	4- to 6-option Likert scales	4 weeks	Physical functioning (1)<br>Role limitations (1)<br>Social functioning (1)<br>Pain (1)<br>Mental health (1)<br>Vitality (1)	0–1<br>0 denotes death<br>100 denotes perfect health	Brazier et al 2002 [39]
Liver disease- and NASH-specific measures						
Chronic Liver Disease Questionnaire (CLDQ)	29	7-option Likert scale	2 weeks	Abdominal symptoms (3)<br>Activity/energy (5)<br>Emotional (8)	1–7<br>0 denotes worse health	Younossi et al 1999 [40]

Instrument/ scale	No. items	Response format	Recall period	Domains (items per domain)	Possible range	Reference
				Fatigue (5)<br>Worry (5)<br>Systematic symptoms (5)	100 denotes better health	
Chronic Liver Disease Questionnaire-NASH (CLDQ-NASH)	36	7-option Likert scale	2 weeks	Abdominal symptoms (3)<br>Activity/energy (5)<br>Emotional health (9)<br>Fatigue (6)<br>Worry (7)<br>Systemic symptoms (6)	1–7<br>0 denotes worse health<br>100 denotes better health	Younossi et al 2017 [41]<br>Younossi et al 2019 [24]
NASH-CHECK (pilot)[§]	31	NR	1 week	Symptoms (10)<br>Activity limitations (8)<br>Emotion and lifestyle (13)	NR	Doward et al 2020 [7]

GERD, gastro-oesophageal reflux disorder; HRQoL, health-related quality of life; MCS, mental component summary; N/A, not applicable; NR, not recorded; PCS, physical component summary; PRO, patient-reported outcome.

*Two items contain seven options (score range: 0, 1a, 1b, 2a, 2b, 3a, 3b) to differentiate between increases and decreases in behaviour; [†]Information shown is for the 2015–2016 version of SPAN questionnaire. An older version of the questionnaire was used in the Hattar et al. (2011) [10] study in the systematic literature review; [‡]Two versions of WPAI are available: (1) General Health and (2) Specific Health Problem; [§]The 31-item pilot version of the NASH-CHECK was included in an interventional phase II study and will undergo psychometric analysis to finalise the structure, explore its measurement properties and responsiveness, and inform the interpretation of scores.

Table S4. Quantitative study quality assessment by EPHPP.

	Study	Selection bias	Study design	Confounders	Blinding	Data collection methods	Withdrawals and drop-outs	Global rating
1	Alt et al 2016 [1]	Strong	Moderate	Weak	Moderate	Moderate	Moderate	Moderate
2	Cook et al 2019 [5]	Strong	Moderate	Weak	Moderate	Moderate	Moderate	Moderate
3	Cook et al 2019 [6]	Strong	Moderate	Weak	Moderate	Moderate	Moderate	Moderate
4	Gholami et al 2018 [9]	Strong	Moderate	Weak	Moderate	Moderate	Moderate	Moderate
5	Hattar et al 2011 [10]	Strong	Moderate	Weak	Moderate	Moderate	Moderate	Moderate
6	Huber et al 2019 [21]	Strong	Moderate	Weak	Moderate	Moderate	Moderate	Moderate
7	Younossi et al 2019 [17]	Moderate	Moderate	Weak	Moderate	Strong	Moderate	Moderate
8	Doward et al 2020 [7]	Moderate	Moderate	Weak	Moderate	Moderate	Moderate	Moderate
9	Elliott et al 2013 [20]	Moderate	Moderate	Weak	Moderate	Moderate	Moderate	Moderate
10	Funuyet-Salas et al 2020 [23]	Moderate	Moderate	Weak	Moderate	Moderate	Moderate	Moderate
11	Geier et al 2021 [8]	Moderate	Moderate	Weak	Moderate	Moderate	Moderate	Moderate
12	O'Hara et al 2020 [14]	Moderate	Moderate	Weak	Moderate	Moderate	Moderate	Moderate
13	Taketani et al 2014 [22]	Moderate	Moderate	Weak	Moderate	Moderate	Moderate	Moderate
14	Chawla et al 2016 [4]	Weak	Moderate	Weak	Moderate	Strong	Moderate	Moderate
15	Balp et al 2019 [3]	Strong	Moderate	Weak	Moderate	Strong	Weak	Weak
16	Noto et al 2014 [12]	Moderate	Moderate	Weak	Moderate	Weak	Strong	Weak
17	Ock et al 2017 [13]	Moderate	Moderate	Weak	Moderate	Weak	Moderate	Weak

EPHPP, Effective Public Health Practice Project.

Table S5. Quality assessment of randomised controlled trials.

	Nikroo et al 2017 [11] (IRCT201104286319N1)	Younossi et al 2018 [16] (NCT02466516)	Armstrong et al 2016 [2] (NCT01237119)	Sanyal et al 2010 [15] (NCT00063622)	Younossi et al 2019* [24] (NCT03053050, NCT03053063)	Younossi et al 2021 [19] (NCT01672866, NCT01672879, NCT03053050, NCT03053063)
Was randomisation carried out appropriately?	Not clear	Yes	Yes	Yes	Yes	Not clear
Was the concealment of treatment allocation adequate?	Not clear	Not clear	Yes	Yes	Yes	Not clear
Were the groups similar at the outset of the study in terms of prognostic factors?	Yes	Not clear	Yes	Yes	Yes	Not clear
Were the care providers, participants and outcome assessors blind to treatment allocation?	Not clear	Yes	Yes	Yes	Yes	Not clear
Were there any unexpected imbalances in drop-outs between groups?	No	No	No	No	No	Not clear
Is there any evidence to suggest that the authors measured more outcomes than they reported?	No	No	No	No	No	Not clear
Did the analysis include an intention-to-treat analysis? If so, was this appropriate and were appropriate methods used to account for missing data?	Yes	Yes	Yes	Yes	Yes	Yes

*Linked to Younossi et al 2020 [18] and Younossi et al 2019 [25]; linked references did not clearly describe results for all categories, but analyses are based on data from the same studies and patient cohort and are therefore assumed to be equivalent to Younossi et al 2019 [24].

Table S6. Patient characteristics across the 23 studies.*

Reference and country	Patient groups (N)	Age, years Mean (SD)	Gender, n (%)	BMI, kg/m^2 Mean (SD)	Diagnosis of NASH	Comorbidities, n (%)
Quantitative studies reporting HRQoL in patients with NASH (N = 15)						
Alt et al 2016 [1]<br>Germany	Non-infectious CLD patients (150)<br>NASH cohort (29)	Median (range): 52 (24–76)	Female: 15 (51.7)<br>Male: 14 (48.3)	NR	Liver biopsy and/or clinical assessment[†]	• Obesity:[‡] 20 (69.0)<br>• T2D: 8 (27.6)<br>• Hypertension: 14 (48.3)<br>• Hyperlipidaemia: 20 (69.0)<br>• Cirrhosis: 10 (34.5)
Balp et al 2019 [3]<br>Europe (France, Germany, Italy, Spain and the UK)	NASH patients (184)<br>Unmatched T2D patients (4783)<br>Unmatched general population (79 267)	**NASH:**<br>54.5 (13.1)<br>**T2D:**<br>62.8 (10.9)<br>**General population:**<br>48.1 (16.5)	**NASH:**<br>Female: 79 (42.9)<br>Male: 105 (57.1)<br>**T2D:**<br>Female: 1635 (34.2)<br>Male: 3148 (65.8)<br>**General population:**<br>Female: 43 937 (55.4)	NR	Self-reported physician diagnosis[†]	**NASH:**<br>• Obesity:[‡] 86 (46.7)<br>• T2D: 42 (22.8)<br>• Hypertension: 91 (49.5)<br>• ≥1 heart or blood condition: 127 (69.0)<br>**T2D:**<br>• Obesity:[‡] 2130 (44.5)<br>• Hypertension: 2686 (56.2)<br>• ≥1 heart or blood condition: 3444 (72.0)<br>**General population:**<br>• Obesity:[‡] 13 313 (16.8)<br>• T2D: 4783 (6.0)<br>• Hypertension: 14 217 (17.9)

Reference and country	Patient groups (N)	Age, years Mean (SD)	Gender, n (%)	BMI, kg/m^2 Mean (SD)	Diagnosis of NASH	Comorbidities, n (%)
			Male: 35 330 (44.6)			• ≥1 heart or blood condition: 25 763 (32.5)
Chawla et al 2016 [4] US	NASH patients (79) Matched US population (2474)	46 (11) (range:19–73)	Female: NR (65) Male: NR (35)	31.4 (range: 19–59)	Liver biopsy revealing >10% steatosis and lobular inflammation with or without fibrosis; Abnormal serum liver tests for >3 months; History of alcohol consumption <40 g/day (men) or <30 g/day (women); No evidence of cirrhosis	• Obesity:[‡] NR (60) • Diabetes: NR (19) • Hypertension: NR (37)
Cook et al 2019 [5] Canada, Germany, UK and US	Confirmed or suspected NASH with F2 or F3 fibrosis stage (166)	52.03 (11.78)	Female: 84 (50.6) Male: 82 (49.4)	NR	Biopsy (n=94) or transient elastography (FibroScan) or ultrasound (UK only)[†]	• Obesity:[§] 114 (68.7) • Diabetes or prediabetes: 88 (53.0) • Hypertension: 80 (48.2) • Dyslipidaemia: 72 (43.4) • CAD: 19 (11.4) • Depression: 26 (15.7) • Sleep apnoea: 25 (15.1)
Elliott et al 2013 [20] UK	ALD and NAFLD patients with	**NAFLD:** 59 (13)	**NAFLD:** Female: 101 (45)	**NAFLD:** NR	**NAFLD:** Biopsy	**NAFLD:** Cirrhosis: 18 (8)

Reference and country	Patient groups (N)	Age, years Mean (SD)	Gender, n (%)	BMI, kg/m² Mean (SD)	Diagnosis of NASH	Comorbidities, n (%)
	steatohepatitis subgroup<br>NAFLD cohort (224)<br>ALD cohort (107)<br>NASH (NR)		Male: 123 (55)			
Funuyet-Salas et al 2020 [23]<br>Spain	NAFLD patients (492)<br>NASH cohort (291)<br>NAFL cohort (201)	**NASH:** 55.49 (11.81)<br>**No NASH:** 54.05 (11.62)	**NASH:**<br>Female: NR (63.4)<br>Male: NR (56.2)<br>**No NASH:**<br>Female: NR (36.6)<br>Male: NR (43.8)	NR	Liver biopsy[†]	NR
Geier et al 2021 [8]<br>France, Germany, US	NASH patients (1216)<br>Biopsy-confirmed cohort (786)<br>**Biopsy-confirmed cohort:**	**Total:** 54.9 (12.3)<br>**Biopsy-confirmed:** 54.6 (11.8)<br>**F1:** 53.6 (12.4)<br>**F2:** 54.7 (11.9)	**Total:**<br>Female: 517 (42.5)<br>Male: 699 (57.5)<br>**Biopsy-confirmed:**<br>Female:	**Total:** 31.7 (6.9)<br>**Biopsy-confirmed:** 31.2 (6.9)<br>**F1:** 30.5 (7.0)<br>**F2:** 30.7 (5.9)	Liver biopsy or phenotypic diagnosis (combination of clinical, laboratory and imaging parameters with physician clinical judgement)[†]	**Total:**<br>• Any comorbidity: 502 (41.3)<br>• Obesity: NR (47.6)[¶]<br>• T2D: NR (59.2)[¶]<br>• Hypertension: NR (48.2)[¶]<br>• Dyslipidaemia: NR (40.4)[¶]<br>• Sleep apnoea: NR (13.3)[¶]

Reference and country	Patient groups (N)	Age, years Mean (SD)	Gender, n (%)	BMI, kg/m² Mean (SD)	Diagnosis of NASH	Comorbidities, n (%)
	No fibrosis (55)<br>F1 (75)<br>F2 (278)<br>F3 (211)<br>F4 (47)<br>Unknown (20)	**F3:** 55.3 (11.0)<br>**F4:** 58.0 (10.1)	343 (43.6)<br>Male: 443 (56.4)<br>**F1:**<br>Female: 85 (48.6)<br>Male: 90 (51.0)<br>**F2:**<br>Female: 116 (41.7)<br>Male: 162 (58.3)<br>**F3:**<br>Female: 99 (46.9)<br>Male: 112 (53.1)<br>**F4:**<br>Female: 15 (31.9)<br>Male: 32 (68.1)	**F3:**<br>32.7 (7.8)<br>**F4:**<br>33.9 (6.9)		• Depression: NR (10.4)¶<br>• CAD: NR (9.6)¶<br>**Biopsy-confirmed:**<br>• Any comorbidity: 296 (37.7)<br>• Obesity: 151 (51.0)¶<br>• T2D: 184 (62.2)¶<br>• Hypertension: 142 (48.0)¶<br>• Dyslipidaemia: 123 (41.6)¶<br>• Sleep apnoea: 42 (14.2)¶<br>• Depression: 34 (11.5)¶<br>• CAD: 17 (5.7)¶<br>**F1:**<br>• Any comorbidity: 53 (30.3)<br>• Obesity: 23 (43.4)¶<br>• T2D: 29 (54.7)¶<br>• Hypertension: 24 (45.3)¶<br>• Dyslipidaemia: 19 (35.8)¶<br>• Sleep apnoea: 4 (7.5)¶<br>• Depression: 5 (9.4)¶<br>• CAD: 2 (3.8)¶<br>**F2:**<br>• Any comorbidity: 92 (33.1)<br>• Obesity: 53 (57.6)¶

Reference and country	Patient groups (N)	Age, years Mean (SD)	Gender, n (%)	BMI, kg/m^2 Mean (SD)	Diagnosis of NASH	Comorbidities, n (%)
						• T2D: 57 (62.0)¶<br>• Hypertension: 38 (41.3)¶<br>• Dyslipidaemia: 39 (42.4)¶<br>• Sleep apnoea: 16 (17.4)¶<br>• Depression: 11 (12.0)¶<br>• CAD: 6 (6.5)¶<br>**F3:**<br>• Any comorbidity: 103 (48.8)<br>• Obesity: 51 (49.5)¶<br>• T2D: 68 (66.0)¶<br>• Hypertension: 54 (52.4)¶<br>• Dyslipidaemia: 51 (49.5)¶<br>• Sleep apnoea: 11 (10.7)¶<br>• Depression: 11 (10.7)¶<br>• CAD: 7 (6.8)¶<br>**F4:**<br>• Any comorbidity: 28 (59.6)<br>• Obesity: 13 (46.4)¶<br>• T2D: 20 (71.4)¶<br>• Hypertension: 15 (53.6)¶<br>• Dyslipidaemia: 9 (32.1)¶<br>• Sleep apnoea: 8 (28.6)¶<br>• Depression: 3 (10.7)¶<br>• CAD: 2 (7.1)¶

Reference and country	Patient groups (N)	Age, years Mean (SD)	Gender, n (%)	BMI, kg/m^2 Mean (SD)	Diagnosis of NASH	Comorbidities, n (%)
Gholami et al 2018 [9] Iran	Suspected NASH patients (332)	42 (13) (range 18–82)	Female: 96 (28.9) Male: 236 (71.1)	31.5 (4.7)	Transabdominal ultrasonography + elevated serum ALT without any other liver diseases†	• Obesity:§ 317 (95.5)<br>• Diabetes: 39 (11.7)<br>• Hypertension: 97 (29.2)<br>• Metabolic syndrome: 144 (43.4)
Hattar et al 2011 [10] US	Hispanic children with NASH and obesity (20)<br>Hispanic children with no evidence of liver disease, lean (17) and obese (20) control groups	**NASH:** 12.2 (2)<br>**Obese:** 11.9 (2)<br>**Lean:** 12.4 (2.1)	**NASH:** Female: 5 (25) Male: 15 (75)<br>**Obese:** Female: 5 (25) Male: 15 (75)<br>**Lean:** Female: 5 (29) Male: 12 (71)	**BMI percentile, % (SD):**<br>**NASH:** 98.05 (1.73)<br>**Obese:** 98.31 (1.42)<br>**Lean:** 48.95 (21.85)	ALT >40 U/L on two separate occasions separated by at least 3 months (90 days apart), and<br>Liver biopsy: ≥5% of hepatocytes with macrovesicular fat, without evidence of other aetiologies for presence of fat and with a pattern of injury consistent with NASH†	NR
Huber et al 2019 [21] Germany, Spain, UK	NAFLD patients (304)<br>NASH cohort (210)<br>NAFL cohort (94)	NR	NR	NR	NASH CRN criteria (liver biopsy revealing NAS ≥5)<br>Alcohol consumption thresholds as per EASL guidelines (<30 g/day for men	**NASH:**<br>• Obesity:‡ 54.9%<br>• T2D: 39.9%<br>**NAFL:**<br>• Obesity:‡ 20.1%<br>• T2D: 11.5%

Reference and country	Patient groups (N)	Age, years Mean (SD)	Gender, n (%)	BMI, kg/m^2 Mean (SD)	Diagnosis of NASH	Comorbidities, n (%)
					and <20 g/day for women)	
Noto et al 2014 [12] Japan	NASH patients (171)<br>NAFL patients (29)<br>Healthy controls (49)	**Average, years:**<br>**NASH:**<br>Male: 45<br>Female: 58<br>**NAFL:**<br>Male: 49<br>Female: 61<br>**Healthy:**<br>Male: 46<br>Female: 54	**NASH**:<br>Male: 93<br>Female: 78<br>**NAFL**:<br>Male: 16<br>Female: 13<br>**Healthy**:<br>Male: 21<br>Female: 28	NR	Liver biopsy revealing ≥5% macrovesicular steatosis with lobular inflammation, ballooning, degeneration and/or perivenular and/or pericellular fibrosis<br>Intake of <140 g of ethanol per week	**Male/female, %:**<br>**NASH:**<br>• Obesity:[#] 77.4/59.0<br>• DM: 25.8/42.3<br>• Hypertension: 32.3/42.3<br>• Dyslipidaemia: 34.4/44.9<br>**NAFL:**<br>• Obesity:[#] 68.8/69.2<br>• DM: 12.5/30.8<br>• Hypertension: 56.3/46.2<br>• Dyslipidaemia: 50.0/69.2<br>**Healthy:**<br>• Obesity:[#] 9.5/3.6<br>• DM: 4.8/3.6<br>• Hypertension: 14.3/3.6<br>• Dyslipidaemia: 14.3/10.7
Ock et al 2017 [13] Korea	Korean general population aged ≥19 years with subset of patients with liver diseases	NR	Female: NR<br>Male: NR	NR	Blood test (such as liver function tests), or an ultrasound[†]	NR

Reference and country	Patient groups (N)	Age, years Mean (SD)	Gender, n (%)	BMI, kg/m² Mean (SD)	Diagnosis of NASH	Comorbidities, n (%)
	including NASH (407)<br>NASH cohort (NR)					
O'Hara et al 2020 [14]<br>USA and Europe (France, Germany, Italy, Spain and the UK)	NASH patients (3754)<br>Provided HRQoL information (767)<br>F0–F2 fibrosis cohort (2604)<br>F3–F4 fibrosis cohort (1150)	**Total:**<br>53 (11.9)<br>**F0–F2:**<br>52 (12.0)<br>**F3–F4:**<br>55 (11.4)	**Total:**<br>Female: 1604 (43)<br>Male: 2150 (57)<br>**F0–F2:**<br>Female: 1111 (43)<br>Male: 1493 (57)<br>**F3–F4:**<br>Female: 493 (43)<br>Male: 657 (57)	**Total:**<br>30.8 (8.7)<br>**F0–F2:**<br>30.8 (9.0)<br>**F3–F4:**<br>30.7 (7.8)	Liver biopsy (NASH with fibrosis), or Clinical biochemistry and/or serum biomarkers indicating advanced fibrosis in patients with metabolic syndrome risk factors, or<br>Imaging techniques indicating advanced fibrosis and/or cirrhosis in patients with metabolic syndrome risk factors†	**Total:**<br>• ≥1 comorbidity: 64%<br>• Obesity: 35%<br>• T2D: 27%<br>• Hypertension: 27%<br>• Dyslipidaemia: 32%<br>• Depression: 8%
Taketani et al 2014 [22]<br>Japan	NAFLD patients (123)<br>NASH cohort (83)<br>NAFL cohort (40)	**NASH:**<br>62 (14–82)<br>**NAFL:**<br>56 (20–78)	**NASH:**<br>Female: 59 (71)<br>Male: 24 (29)<br>**NAFL:**<br>Female: 17 (43)	**NASH:**<br>27.3 (IQR: 16.6–41.0)<br>**NAFL:**<br>26.6 (IQR: 18.9–43.4)	Liver biopsy revealing ≥5% steatosis with lobular inflammation and ballooning degeneration, with or without Mallory-	**NASH:**<br>• Obesity:# 60 (72)<br>• T2D: 42 (51)<br>• Hypertension: 38 (46)<br>• Dyslipidaemia: 34 (41)<br>**NAFL:**<br>• Obesity:# 27 (68)

Reference and country	Patient groups (N)	Age, years Mean (SD)	Gender, n (%)	BMI, kg/m^2 Mean (SD)	Diagnosis of NASH	Comorbidities, n (%)
			Male: 23 (58)		Denk body or fibrosis<br>Alcohol consumption ≤20 g/day	• T2D: 13 (33)<br>• Hypertension: 10 (25)<br>• Dyslipidaemia: 12 (30)
Younossi et al 2019 [17]<br>Global	Patients with NASH and advanced fibrosis** (1338)<br>Matched patients with CHC (1338)	**NASH:**<br>56.9 (8.9)<br>**CHC:**<br>57.6 (8.2)	**NASH:**<br>Female: 710 (53.1)<br>Male: 628 (46.9)<br>**CHC:**<br>Female: 710 (53.1)<br>Male: 628 (46.9)	**NASH:**<br>33.7 (73.8)<br>**CHC:**<br>27.6 (5.5)	Liver biopsy†	**NASH:**<br>• T2D: 988 (73.8)<br>• Psychiatric comorbidities: 543 (40.6)<br>• Anxiety: 260 (19.4)<br>• Depression or mood disorders: 339 (25.3)<br>**CHC:**<br>• T2D: 215 (16.1)<br>• Psychiatric comorbidities: 533 (39.8)<br>• Anxiety: 227 (17.0)<br>• Depression or mood disorders: 324 (24.2)
Qualitative studies reporting HRQoL in patients with NASH (N = 2)						
Cook et al 2019 [6]<br>UK, US	NASH patients with mild (stage F1) to severe (F3) fibrosis (16)<br>US cohort (8)<br>UK cohort (8)	**US:**<br>42.6 (34–49)<br>**UK:**<br>47.4 (27–70)	**US:**<br>Female: 4<br>Male: 4<br>**UK:**<br>Female: 6<br>Male: 2	NR	Liver biopsy, or Blood test, ultrasound, CT, and/or MRI†	**US:**<br>• Obesity:§ 7 (88)<br>• Diabetes: 6 (75)<br>**UK:**<br>• Obesity:§ 5 (63)<br>• Diabetes: 3 (38)

Reference and country	Patient groups (N)	Age, years Mean (SD)	Gender, n (%)	BMI, kg/m^2 Mean (SD)	Diagnosis of NASH	Comorbidities, n (%)
Doward et al 2020 [7]<br>US	Non-cirrhotic NASH patients (35)<br>CE cohort (23)<br>CD cohort (20)<br>*8 patients participated in both groups*	**CE:**<br>55.9 (10.0; range: 31.0–73.0)<br>**CD:**<br>50.6 (10.4; range: 30.0–68.0)	**CE group:**<br>Female: 18 (78.3)<br>Male: 5 (21.7)<br>**CD group:**<br>Female: 11 (55.0)<br>Male: 9 (45.0)	**CE:**<br>33.5 (5.4; range: 25.8–45.4)<br>**CD:**<br>35.9 (6.3; range: 26.2–54.9)	NASH CRN criteria (liver biopsy revealing NAS ≥5), or phenotypic diagnosis (i.e., ALT ≥60 IU/L for males or ≥40 IU/L for females; BMI ≥23 kg/m^2 in Asian individuals or ≥27 kg/m^2 in non-Asian individuals)<br>Average alcohol consumption ≤20 g/day in females and ≤30 g/day in males for a period of >3 consecutive months within 1 year prior to study	**CE group:**<br>• Obesity:‡ 16 (69.6)<br>• T2D: 14 (60.9)<br>• Hypertension: 16 (69.6)<br>• Hyperlipidaemia: 12 (52.2)<br>• Depression: 7 (30.4)<br>• Asthma: 5 (21.7)<br>• Anxiety: 4 (17.4)<br>**CE group:**<br>• Obesity:‡ 18 (90.0)<br>• T2D: 14 (70.0)<br>• Hypertension: 13 (65.0)<br>• Hyperlipidaemia: 11 (55.0)<br>• Depression: 6 (30.0)<br>• Asthma: 5 (25.0)<br>• Anxiety: 4 (20.0)
Interventional studies reporting HRQoL in patients with NASH (N = 6)						
Armstrong et al 2016 [2]<br>UK	NASH patients with overweight (52)<br>Liraglutide 1.8 mg QD cohort (26)	**Liraglutide:**<br>50 (11)<br>**Placebo:**<br>52 (12)	**Liraglutide:**<br>Female: 8 (31)<br>Male: 18 (69)<br>**Placebo:**	**Liraglutide:**<br>34.2 (4.7)<br>**Placebo:**<br>37.7 (6.2)	Liver biopsy revealing >5% macrovesicular steatosis, hepatocyte ballooning and lobular inflammation	**Liraglutide:**<br>• T2D: 9 (35)<br>• Hypertension: 15 (58)<br>• Hyperlipidaemia: 9 (35)<br>• CVD: 0 (0)<br>• Hypothyroidism: 3 (12)

Reference and country	Patient groups (N)	Age, years Mean (SD)	Gender, n (%)	BMI, kg/m^2 Mean (SD)	Diagnosis of NASH	Comorbidities, n (%)
	Placebo cohort (26)		Female: 13 (50)<br>Male: 13 (50)		Alcohol consumption ≤20 g/day for women or ≤30 g/day for men)	**Placebo:**<br>• T2D: 8 (31)<br>• Hypertension: 14 (54)<br>• Hyperlipidaemia: 7 (27)<br>• CVD: 3 (12)<br>• Hypothyroidism: 4 (15)
Nikroo et al 2017 [11]<br>Iran	Male NASH patients (25)<br>Calorie-restricted diet and aerobic exercises cohort (12)<br>Calorie-restricted-diet alone cohort (13)	**Diet and exercise:** 38.67 (7.36)<br>**Diet alone:** 35.64 (9.22)	Female: 0<br>Male: 25 (100)	**Diet and exercise:** 30.37 (4.5)<br>**Diet alone:** 32.58 (6.62)	Ultrasound (graded based on hepatic-renal echo contrast, brightness, deep attenuation and vascular blurring)<br>Historical alcohol consumption ≤20 g/day for women or ≤30 g/day for men)	NR
Sanyal et al 2010 [15]<br>US	NASH patients without diabetes (247)<br>Placebo cohort (83)<br>Vitamin E 800 IU QD (natural form) cohort (84)	**Placebo:** 45.4 (11.2)<br>**Vitamin E:** 46.6 (12.1)<br>**Pioglitazone:** 47.0 (12.6)	**Placebo:** Female: NR (58)<br>Male: NR (42)<br>**Vitamin E:** Female: NR (62)<br>Male: NR (38)	**Placebo:** 35 (7)<br>**Vitamin E:** 34 (7)<br>**Pioglitazone:** 34 (6)	Liver biopsy revealing definite or possible steatohepatitis with NAS ≥5, or definite steatohepatitis (confirmed by two pathologists) with NAS of 4, including a score of ≥1 for	NR

Reference and country	Patient groups (N)	Age, years Mean (SD)	Gender, n (%)	BMI, kg/m^2 Mean (SD)	Diagnosis of NASH	Comorbidities, n (%)
	Pioglitazone 30 mg QD cohort (80)		**Pioglitazone:** Female: NR (59) Male: NR (41)		hepatocellular ballooning<br>Alcohol consumption ≤20 g/day for women or ≤30 g/day for men) for ≥3 consecutive months during previous 5 years	
Younossi et al 2018 [16]<br>Canada and US	NASH F2 fibrosis (47)<br>NASH F3 fibrosis (25)	**F2:** 55.5 (9.6)<br>**F3:** 51.6 (10.2)	**F2:** Female: 33 (70.2) Male: 14 (29.8)<br>**F3:** Female: 17 (68.0) Male: 8 (32.0)	**F2:** 35.2 (9.0)<br>**F3:** 33.4 (4.7)	Liver biopsy revealing NAS ≥5 with score ≥1 for each of its components (steatosis, hepatocyte ballooning, lobular inflammation)<br>Alcohol consumption ≤21 oz/week for men or ≤14 oz/week for women (Loomba et al 2018) [42]	**Stage 2 fibrosis:**<br>• T2D: 37 (78.7)<br>• Hypertension: 40 (85.1)<br>• Drug-treated hypertension: 35 (74.5)<br>• Anxiety or panic disorders: 14 (29.8)<br>• Depression or mood disorders: 12 (25.5)<br>• Clinically overt fatigue: 8 (17.0)<br>• Insomnia or sleep disorders: 7 (14.9)<br>**Stage 3 fibrosis:**<br>• T2D: 14 (56.0)<br>• Hypertension: 15 (60.0)<br>• Drug-treated hypertension: 13 (52.0)<br>• Anxiety or panic disorders: 6 (24.0)

**Reference and country**	**Patient groups (N)**	**Age, years Mean (SD)**	**Gender, n (%)**	**BMI, kg/m^2 Mean (SD)**	**Diagnosis of NASH**	**Comorbidities, n (%)**
						• Depression or mood disorders: 4 (16.0)<br>• Clinically overt fatigue: 6 (24.0)<br>• Insomnia or sleep disorders: 7 (28.0)
Younossi et al 2019 [24]<br>(Linked to Younossi et al 2020 [18] and Younossi et al 2019 [25])<br>Global	Biopsy-proven NASH with advanced fibrosis** (1667[††])	57.8 (8.8)	Female: 994 (59.6)<br>Male: 673 (40.4)	33.5 (6.6)	Liver biopsy revealing NAS score of grade ≥1 steatosis, hepatocyte ballooning and lobular inflammation<br>No current or recent alcohol abuse[†]	• History of T2D: 1157 (69.4)<br>• Cirrhosis: 870 (52.2)<br>• History of psychiatric disorders: 696 (41.8)<br>• History of clinically overt fatigue: 164 (9.8)
Younossi et al 2021 [19]<br>Global	Biopsy-proven NASH with advanced fibrosis** (2154)<br>Bridging fibrosis cohort (1021)<br>CC cohort (1133)	**Total:** 57.1 (8.9)<br>**Bridging fibrosis:** 56.5 (9.2)<br>**CC:** 57.6 (8.4)	**Total:** Female: 1300 (60.4) Male: 854 (39.6)<br>**Total:** Female: 590 (57.8) Male: 431 (42.2)<br>**Total:** Female:	**Total:** 33.7 (6.6)<br>**Bridging fibrosis:** 33.5 (6.5)<br>**CC:** 33.8 (6.7)	Liver biopsy consistent with NASH based on NAS score<br>No recent excessive alcohol intake[†]	**Diabetes mellitus:**<br>• Total: 1555 (72.2)<br>• Bridging fibrosis: 706 (69.1)<br>• CC: 849 (74.9)

Reference and country	Patient groups (N)	Age, years Mean (SD)	Gender, n (%)	BMI, kg/m^2 Mean (SD)	Diagnosis of NASH	Comorbidities, n (%)
			710 (62.7) Male: 423 (37.3)			

ADL, activities of daily living; ALT, alanine aminotransferase; AS, abdominal symptoms; BMI, body mass index; CAD, coronary artery disease; CC, compensated cirrhosis; CD, cognitive debriefing; CE, concept elicitation; CHC, chronic hepatitis C; CLD, chronic liver disease; CRN, Clinical Research Network; CT, computed tomography; CVD, cardiovascular disease; DM, diabetes mellitus; F1–4, fibrosis stage 1–4; HRQoL, health-related quality of life; IHC, immunohistochemistry; IQR, interquartile range; MRI, magnetic resonance imaging; NAFL, non-alcoholic fatty liver; NAFLD, non-alcoholic fatty liver disease; NAS, NAFLD Activity Score; NASH, non-alcoholic steatohepatitis; NFS, NAFLD fibrosis score; NR, not reported; QD, once daily; SD, standard deviation; T2D, type 2 diabetes.

*Data presented are for NASH populations unless otherwise specified; †No information on participant alcohol consumption provided; ‡Obesity defined as BMI >30 kg/m^2 or ≥30 kg/m^2; §Obesity criteria not defined; ¶Proportion of patients with any comorbidity; #BMI >25 kg/m^2; **Bridging fibrosis (F3) or compensated cirrhosis (F4); ††Outcomes reported in linked publications (Younossi et al 2020 [18]; Younossi et al 2019 [25]) included 1669 and 1667 participants, respectively.

Table S7. Instruments/scales used to assess HRQoL in included studies.

Study	SF-36	CLDQ	Symptoms	WPAI	EQ-5D-5L	AIS	CLDQ-NASH	EQ-5D	FSSG	PHAQ	Physical activity	SF-12	SF-6D	SPAN	Utility (VAS, SG)	HADS	BDI-II
Armstrong et al 2016 [2]	x*																
Balp et al 2019 [3]	x*		x					x					x				
Chawla et al 2016 [4]	x	x															
Nikroo et al 2017 [11]	x																
Sanyal et al 2010 [15]	x																
Younossi et al 2018 [16]	x	x		x													
Younossi et al 2019 [17]	x	x		x													
Younossi et al 2020† [18]	x			x			x	x					x				
Younossi et al 2021 [19]	x			x			x	x					x				
Alt et al 2016 [1]		x‡															
Huber et al 2019 [21]		x															
Geier et al 2020 [8]		x		x	x												
O'Hara et al 2020 [14]				x	x		x§										
Funuyet-Salas et al 2020 [23]							x§					x				x	x
Cook et al 2019 [5]			x		x												
Cook et al 2019 [6]			x														
Doward et al 2020 [7]			x														
Taketani et al 2014 [22]						x			x								
Elliott et al 2013 [20]										x							
Noto et al 2014 [12]											x						
Gholami et al 2018 [9]												x					
Hattar et al 2011 [10]														x			

Study	SF-36	CLDQ	Symptoms	WPAI	EQ-5D-5L	AIS	CLDQ-NASH	EQ-5D	FSSG	PHAQ	Physical activity	SF-12	SF-6D	SPAN	Utility (VAS, SG)	HADS	BDI-II
Ock et al 2017 [13]															x		

AIS, Athens Insomnia Scale; BDI-II, Beck Depression Inventory®-II; CLDQ, Chronic Liver Disease Questionnaire; EQ−5D, EuroQol-5D; EQ-5D-5L, EuroQol-5D-5 level; FSSG, frequency scale for the symptoms of gastro-oesophageal reflux disorder; HADS, Hospital Anxiety and Depression Scale; HRQoL, health-related quality of life; NASH, non-alcoholic steatohepatitis; PHAQ, Patient-Reported Outcome Measurement Information System Health Assessment Questionnaire; SF-12, Short Form-12; SF-36, Short Form-36; SF-6D, Short Form-6 Dimension; SG, standard gamble; SPAN, School Physical Activity and Nutrition; VAS, visual analogue scale; WPAI, Work Productivity and Activity Impairment Questionnaire.

*SF-36v2 specified in Armstrong et al 2016 [2] and Balp et al 2019 [3]; †Linked to Younossi et al 2019 [24] and Younossi et al 2019 [25] (Younossi et al 2020 [18] and 2019 [25] used CLDQ-NASH, SF-36, WPAI, EQ-5D and SF-6D, Younossi et al 2019 [24] used CLDQ-NASH and SF-36); ‡The German version of CLDQ; §CLDQ-NAFLD.

Supplementary references

1. Alt Y, Grimm A, Schlegel L, Grambihler A, Kittner JM, Wiltink J, et al. The impact of liver cell injury on health-related quality of life in patients with chronic liver disease. PLoS One 2016;11:e0151200.
2. Armstrong MJ, Gaunt P, Aithal GP, Barton D, Hull D, Parker R, et al. Liraglutide safety and efficacy in patients with non-alcoholic steatohepatitis (LEAN): a multicentre, double-blind, randomised, placebo-controlled phase 2 study. Lancet 2016;387:679–690.
3. Balp MM, Krieger N, Przybysz R, Way N, Cai J, Zappe D, et al. The burden of non-alcoholic steatohepatitis (NASH) among patients from Europe: a real-world patient-reported outcomes study. JHEP Rep 2019;1:154–161.
4. Chawla KS, Talwalkar JA, Keach JC, Malinchoc M, Lindor KD, Jorgensen R. Reliability and validity of the Chronic Liver Disease Questionnaire (CLDQ) in adults with non-alcoholic steatohepatitis (NASH). BMJ Open Gastroenterol 2016;3:e000069.
5. Cook N, Geier A, Schmid A, Hirschfield G, Kautz A, Schattenberg JM, et al. The patient perspectives on future therapeutic options in NASH and patient needs. Front Med (Lausanne) 2019;6:61.
6. Cook NS, Nagar SH, Jain A, Balp MM, Maylander M, Weiss O, et al. Understanding patient preferences and unmet needs in non-alcoholic steatohepatitis (NASH): insights from a qualitative online bulletin board study. Adv Ther 2019;36:478–491.
7. Doward LC, Balp M, Twiss J, Slota C, Cryer D, Brass CA, et al. Development of a patient-reported outcome measure for non-alcoholic steatohepatitis (NASH-CHECK): results of a qualitative study. Patient 2021;14:533–543.
8. Geier A, Rinella ME, Balp MM, McKenna SJ, Brass CA, Przybysz R, et al. Real-world burden of nonalcoholic steatohepatitis. Clin Gastroenterol Hepatol 2021;19:1020–1029.e7.

9. Gholami A, Zamani F, Hosseini B, Sharafkhani R, Maadi M, Jahromi ZM, et al. Metabolic syndrome is associated with health-related quality of life in suspected patients with nonalcoholic steatohepatitis. Med Princ Pract 2018;27:166–172.
10. Hattar LN, Wilson TA, Tabotabo LA, O'Brian Smith E, Abrams SH. Physical activity and nutrition attitudes in obese Hispanic children with non-alcoholic steatohepatitis. World J Gastroenterol 2011;17:4396–4403.
11. Nikroo H, Nematy M, Hosseini SRA, Sima HR, Razmpour F. How does addition of regular aerobic exercises, influence the efficacy of calorie-restricted diet in patients with non-alcoholic steatohepatatis (NASH)? Hepat Mon 2017;17:e45339.
12. Noto H, Tokushige K, Hashimoto E, Taniai M, Shiratori K. Questionnaire survey on lifestyle of patients with nonalcoholic steatohepatitis. J Clin Biochem Nutr 2014;55:191–195.
13. **Ock M, Lim SY**, Lee H-J, Kim S-H, Jo M-W. Estimation of utility weights for major liver diseases according to disease severity in Korea. BMC Gastroenterol 2017;17:103.
14. O'Hara J, Finnegan A, Dhillon H, Ruiz-Casas L, Pedra G, Franks B, et al. Cost of non-alcoholic steatohepatitis in Europe and the USA: the GAIN study. JHEP Rep 2020;2:100142.
15. Sanyal AJ, Chalasani N, Kowdley KV, McCullough A, Diehl AM, Bass NM, et al. Pioglitazone, vitamin E, or placebo for nonalcoholic steatohepatitis. N Engl J Med 2010;362:1675–1685.
16. Younossi ZM, Stepanova M, Lawitz E, Charlton M, Loomba R, Myers RP, et al. Improvement of hepatic fibrosis and patient-reported outcomes in non-alcoholic steatohepatitis treated with selonsertib. Liver Int 2018;38:1849–1859.
17. Younossi ZM, Stepanova M, Lawitz EJ, Reddy KR, Wong VW-S, Mangia A, et al. Patients with nonalcoholic steatohepatitis experience severe impairment of health-related quality of life. Am J Gastroenterol 2019;114:1636–1641.

18. Younossi ZM, Wong VW, Anstee QM, Romero-Gomez M, Trauner MH, Harrison SA, et al. Fatigue and pruritus in patients with advanced fibrosis due to nonalcoholic steatohepatitis: the impact on patient-reported outcomes. Hepatol Commun 2020;4:1637–1650.
19. Younossi ZM, Anstee QM, Wai-Sun Wong V, Trauner M, Lawitz EJ, Harrison SA, et al. The association of histologic and noninvasive tests with adverse clinical and patient-reported outcomes in patients with advanced fibrosis due to nonalcoholic steatohepatitis. Gastroenterology 2021;160:1608–1619.e13.
20. Elliott C, Frith J, Day CP, Jones DEJ, Newton JL. Functional impairment in alcoholic liver disease and non-alcoholic fatty liver disease is significant and persists over 3 years of follow-up. Dig Dis Sci 2013;58:2383–2391.
21. Huber Y, Boyle M, Hallsworth K, Tiniakos D, Straub BK, Labenz C, et al. Health-related quality of life in nonalcoholic fatty liver disease associates with hepatic inflammation. Clin Gastroenterol Hepatol 2019;17:2085–2092.e1.
22. Taketani H, Sumida Y, Tanaka S, Imajo K, Yoneda M, Hyogo H, et al. The association of insomnia with gastroesophageal reflux symptoms in biopsy-proven nonalcoholic fatty liver disease. J Gastroenterol 2014;49:1163–1174.
23. Funuyet-Salas J, Pérez-San-Gregorio MÁ, Martín-Rodríguez A, Romero-Gómez M. Psychological biomarkers and fibrosis: an innovative approach to non-alcoholic fatty liver disease. Front Med (Lausanne) 2020;7:585425.
24. Younossi ZM, Stepanova M, Younossi I, Racila A. Validation of chronic liver disease questionnaire for nonalcoholic steatohepatitis in patients with biopsy-proven nonalcoholic steatohepatitis. Clin Gastroenterol Hepatol 2019;17:2093–2100.e3.
25. Younossi ZM, Stepanova M, Anstee QM, Lawitz EJ, Wong VW-S, Romero-Gomez M, et al. Reduced patient-reported outcome scores associate with level of fibrosis in patients with nonalcoholic steatohepatitis. Clin Gastroenterol Hepatol 2019;17:2552–2560.e10.

26. Soldatos CR, Dikeos DG, Paparrigopoulos TJ. Athens Insomnia Scale: validation of an instrument based on ICD-10 criteria. J Psychosom Res 2000;48:555–560.
27. Beck AT, Steer RA, Brown GK. Beck Depression Inventory®-2. San Antonio, TX, USA: Psychological Corporation; 1996.
28. Kusano M, Shimoyama Y, Sugimoto S, Kawamura O, Maeda M, Minashi K, et al. Development and evaluation of FSSG: frequency scale for the symptoms of GERD. J Gastroenterol 2004;39:888–891.
29. Zigmond AS, Snaith RP. The Hospital Anxiety and Depression Scale. Acta Psychiatr Scand 1983;67:361–370.
30. Stern A. The Hospital Anxiety and Depression Scale. Occup Med (Lond) 2014;64:393–394.
31. Fries JF, Cella D, Rose M, Krishnan E, Bruce B. Progress in assessing physical function in arthritis: PROMIS short forms and computerized adaptive testing. J Rheumatol 2009;36:2061–2066.
32. Michael & Susan Dell Center for Healthy Living (n.d.). School Physical Activity and Nutrition survey (SPAN). Available at: go.uth.edu/SPAN. Accessed 10 February 2021.
33. Ware J, Kosinski M, Keller SD. A 12-item Short-Form Health Survey: construction of scales and preliminary tests of reliability and validity. Med Care 1996;34:220–233.
34. Maruish, ME (Editor). 2011. User's manual for the SF-36v2 health survey, 3rd ed. Lincoln: QualityMetric Incorporated.
35. RAND Health Care (n.d.). 36-item Short Form Survey (SF-36). Available at: https://www.rand.org/health-care/surveys_tools/mos/36-item-short-form.html. Accessed 10 February 2021.
36. Reilly MC, Zbrozek AS, Dukes EM. The validity and reproducibility of a work productivity and activity impairment instrument. Pharmacoeconomics 1993;4:353–365.

37. Reilly Associates (2019). WPAI general information. Available at: http://www.reillyassociates.net/WPAI_General.html. Accessed 10 February 2021.

38. EuroQol (n.d.). EQ-5D. Available at: https://euroqol.org/eq-5d-instruments/. Accessed 10 February 2021.

39. Brazier J, Roberts J, Deverill M. The estimation of a preference-based measure of health from the SF-36. J Health Econ 2002;21:271–292.

40. Younossi ZM, Guyatt G, Kiwi M, Boparai N, King D. Development of a disease specific questionnaire to measure health related quality of life in patients with chronic liver disease. Gut 1999;45:295–300.

41. Younossi ZM, Stepanova M, Henry L, Racila A, Lam B, Pham HT, et al. A disease-specific quality of life instrument for non-alcoholic fatty liver disease and non-alcoholic steatohepatitis: CLDQ-NAFLD. Liver Int 2017;37:1209–1218.

42. Loomba R, Lawitz E, Mantry PS, Jayakumar S, Caldwell SH, Arnold H, et al. The ASK1 inhibitor selonsertib in patients with nonalcoholic steatohepatitis: a randomized, phase 2 trial. Hepatology 2018;67:549–559.
